# Efficacy and safety of ropeginterferon in myeloproliferative neoplasms: a systematic review and meta-analysis

**DOI:** 10.3389/fphar.2026.1841557

**Published:** 2026-09-23

**Authors:** Aadhila Abbas Manthiri, Leen Haj Saleh, Marrita Rabadi, Ayman Dalol, Rowan Mesilhy, Usra Elshaikh, Abdulrahman F. Al-Mashdali, Mohammed Abdulgayoom, Shehab F. Mohamed

**Affiliations:** 1 College of Pharmacy, Qatar University, Doha, Qatar; 2 College of Medicine, Qatar University, Doha, Qatar; 3 Department of Internal medicine, Hamad Medical Corporation, Doha, Qatar; 4 Department of Public Health, College of Health Sciences, QU Health, Qatar University, Doha, Qatar; 5 Department of Hematology, National Center for Cancer Care and Research, Hamad Medical Corporation, Doha, Qatar

**Keywords:** ropeginterferon alfa-2b, efficacy, hematological response, safety, myeloproliferative neoplasms, polycythemia vera, essential thrombocythemia, myelofibrosis

## Abstract

**Background:**

Ropeginterferon alfa-2b (ropegIFN-α2b or ropeginterferon) has emerged as a therapeutic option for patients with myeloproliferative neoplasms, particularly polycythemia vera (PV), with potential disease-modifying effects. This systematic review aimed to evaluate the efficacy and safety of ropeginterferon across clinical and real-world studies.

**Methods:**

A systematic search of PubMed, Embase, and Web of Science was conducted to identify studies reporting clinical outcomes of ropeginterferon in patients with MPNs. An additional PubMed search was performed to capture recently published studies. Eligible studies were screened according to predefined inclusion criteria. Data on hematologic, molecular, clinical, and safety outcomes were extracted and narratively synthesized. A proportional meta-analysis was conducted for selected outcomes, including complete hematologic response (CHR), partial molecular response (PMR), and grade ≥3 cytopenias.

**Results:**

A total of 18 studies involving 1,836 patients were included. Pooled analysis demonstrated that CHR rates increased over time, from 26% at 3 months to 59% at 12 months, with an overall pooled estimate of 56% (95% CI: 46%–66%). PMR was achieved in 37% of patients (95% CI: 20%–59%), although with substantial heterogeneity. Molecular responses were supported by consistent reductions in *JAK2* allele burden across studies. Clinical outcomes showed improvements in spleen size and symptom burden in several cohorts. Ropeginterferon was generally well tolerated. The pooled proportion of grade ≥3 cytopenias was 3% (95% CI: 1%–10%), and severe hepatotoxicity was uncommon, typically occurring in ≤5% of patients. Most adverse events were low grade, with infrequent treatment discontinuations.

**Conclusion:**

RopegIFN-α2b demonstrates favorable efficacy and safety profiles in patients with MPNs, especially in PV, with evidence of progressive hematologic and molecular responses over time. These findings support its role as a potentially disease-modifying therapy, although further standardized and long-term studies, focusing on ET and MF, are needed.

**Systematic Review Registration:**

https://www.crd.york.ac.uk/PROSPERO/view/CRD420261337306, identifier CRD420261337306.

## 1 Introduction

Philadelphia chromosome–negative myeloproliferative neoplasms (MPNs) are clonal hematopoietic stem cell disorders driven by somatic mutations in Janus kinase 2 (*JAK2*), calreticulin (*CALR*), or myeloproliferative leukemia virus oncogene (*MPL*). The classical entities: polycythemia vera (PV), essential thrombocythemia (ET), and primary myelofibrosis (MF) are defined by integrated clinical, morphologic, and molecular criteria. These disorders share a propensity for thrombohemorrhagic complications, symptomatic burden, and long-term risk of transformation to MF or acute myeloid leukemia (Barbui et al., 2018a).

The primary therapeutic goal in PV and ET is reduction of vascular risk. In PV, the importance of hematocrit control was established by the CYTO-PV randomized trial that demonstrated that maintaining hematocrit <45% significantly reduced the composite of cardiovascular death and major thrombosis compared with a less stringent target (Marchioli et al., 2013). Hydroxyurea (HU) has served as the standard cytoreductive agent based on its accessibility and reliable blood count control. However, it is limited by clinically meaningful rates of intolerance or resistance and lacks the capacity to modify underlying disease biology (Marchetti et al., 2022).

Interferon-α (IFN-α), unlike HU, preferentially targets the malignant clone and can induce reductions in driver mutation allele burden, supporting a disease-modifying mechanism of action (Mascarenhas et al., 2022). Conventional interferon-α formulations, including pegylated interferon alfa-2a (Pegasys®) have long served as an alternative cytoreductive therapy, particularly in younger patients and those intolerant of hydroxyurea (Marchetti et al., 2022). However, their use has been limited by frequent dosing schedules, treatment-related adverse effects, and variable tolerability, which may compromise long-term adherence (Mascarenhas et al., 2022). Ropeginterferon alfa-2b (RopegIFN-α2b; hereafter referred to as ropeginterferon) was subsequently developed as a novel mono-pegylated proline-IFN-α2b with an extended half-life that enables less frequent administration while maintaining sustained therapeutic activity. It was approved by the European Medicines Agency in February 2019 (European Medicines Agency, 2018) and by the United States Food and Drug Administration (FDA) in November 2021 (Food and Drug Administration, 2025), making it the first interferon formulation approved specifically for PV. The clinical evidence base for ropeginterferon spans both PV and ET across the risk spectrum. In low-risk PV, it demonstrated superiority over phlebotomy alone in maintaining hematocrit on target, with additional improvements in leukocyte and platelet control (Barbui et al., 2021). In cytoreduction-requiring PV, long-term data from the PROUD-PV and CONTINUATION-PV trials showed higher rates of complete hematologic and molecular response compared with HU, and significantly superior event-free survival over 6 years of follow-up (Gisslinger et al., 2020; Gisslinger et al., 2023). Evidence has since extended to ET, where the SURPASS-ET phase 3 trial demonstrated superior durable response rates over anagrelide in HU-intolerant or HU-resistant patients with leukocytosis, alongside a more favorable safety profile (Mesa et al., 2025).

Yet the full clinical profile of ropeginterferon in real-world practice remains incompletely characterized; real-world data are necessary to capture dosing patterns, adherence, discontinuation rate, and effectiveness in patient populations underrepresented in controlled settings, although such data carry inherent limitations including confounding and non-standardized endpoint ascertainment. A rigorous systematic review and meta-analysis is therefore timely to quantify the magnitude and durability of ropeginterferon’s benefit across hematologic, molecular, and clinical outcomes, and to synthesize safety data in a manner that supports clinical practice and guideline development.

## 2 Methodology

### 2.1 Study design and protocol registration

This study is a systematic review and meta-analysis of studies performed in accordance with the Preferred Reporting Items for Systematic Reviews and Meta-Analyses (PRISMA) guidelines (Page et al., 2021). The study protocol was registered in the International Prospective Register of Systematic Reviews (PROSPERO) with registration ID (CRD420261337306) (Supplementary Table S1).

### 2.2 Eligibility criteria

We included studies that were primary research articles reporting original data, including randomized controlled trials, prospective or retrospective cohort studies, observational studies, and clinical trials. We included studies enrolling patients of any age with a diagnosis of MPNs, including PV, ET, or MF. The intervention of interest was ropegIFN-α2b, including studies referring to the agent under alternative names such as ropeginterferon α-2b, ropeginterferon, P1101, or its trade name: BESREMI®, administered either as monotherapy or in combination with other treatments. Studies were required to report at least one outcome related to efficacy, safety, quality of life, or molecular response.

Only studies published in English between 2020 and 2025 were eligible. Studies were excluded if they were reviews, editorials, commentaries, conference abstracts, secondary analyses, veterinary studies, preclinical or *in vitro* studies, if they evaluated interferon formulations other than ropeginterferon, or if they involved disease conditions outside the scope of MPNs, such as hepatitis C or other hematological malignancies.

### 2.3 Outcomes

The primary efficacy outcome of this review was complete hematologic response (CHR), defined according to the criteria used in each individual study. Secondary efficacy outcomes included partial hematologic response, overall response rate, hematocrit control, freedom from phlebotomy in PV cohorts, molecular responses (complete and partial molecular response, including changes in *JAK2* allele burden), duration of response (DOR), spleen size reduction, symptom improvement, and survival-related outcomes, including progression-free survival (PFS), event-free survival (EFS), and overall survival (OS). Disease progression to MF or acute myeloid leukemia (AML) was also evaluated when reported. Safety outcomes included the incidence of grade ≥3 cytopenias, grade ≥3 hepatotoxicity, grade ≥3 flu-like adverse events, other treatment-related adverse events, treatment discontinuation rates and reasons for discontinuation, and treatment-related mortality.

### 2.4 Search strategy and study selection

A systematic search of PubMed, EMBASE, and Web of Science was conducted for relevant articles published up to 17 October 2025, followed by an updated search in PubMed covering the period from 17 October 2025 to 11 March 2026. Medical subject headings (MeSH) and keyword searches such as (“Myeloproliferative Neoplasm” OR “Polycythemia Vera” OR “Essential Thrombocytopenia” OR “Myelofibrosis”) AND (“Ropeginterferon”) to identify the relevant studies. The full list of keywords and search strategies for the different databases can be found in the Supplementary Material (Search Strategy).

The retrieved articles were uploaded to Rayyan systematic review management website (Ouzzani et al., 2016) for duplication removal and screening. Within Rayyan, preliminary screening of titles and abstracts was conducted independently by two pairs of reviewers: A.M. and R.M., who screened 135 records, and A.D. and M.R., who screened 134 records. Any discrepancies identified during this phase were resolved through discussion between the reviewers, and, when consensus could not be reached, a third reviewer was consulted. Full-text screening was subsequently performed independently by two reviewer pairs (L.H. and R.M., and A.D. and M.R.), with 55 articles assessed by each pair. Disagreements at the full-text stage were resolved using the same consensus-based approach, with involvement of a third reviewer when necessary.

### 2.5 Data extraction

For each included study, two pairs of reviewers (L.H. and A.D., and A.M. and R.M.) independently extracted data using a standardized data extraction sheet created in Microsoft Excel. Reviewers were blinded to each other’s extracted data during this process. Extracted variables included study characteristics (author, year of publication, country, and study design), participant characteristics (age, male %, total sample size, number of participants per arm, follow up duration, disease type, MF PV or ET risk stratification, prior treatments of first line, molecular status, mean baseline allele burden, mean baseline spleen size), intervention details (monotherapy or combination, ropeginterferon schedule, duration of therapy), efficacy outcomes, safety outcomes. Following completion of the extraction, the two data extraction sheets were compared, and discrepancies were reviewed and resolved by a third reviewer (M.R.). Consensus was reached through discussion to ensure accuracy and consistency of the extracted data prior to analysis.

### 2.6 Risk of bias assessment

Methodological quality and risk of bias were assessed independently by the same reviewers involved in data extraction. Randomized controlled trials were evaluated using the RoB 2 tool (Sterne et al., 2019), while non-randomized interventional studies were assessed using the ROBINS-I tool (Sterne et al., 2016). Observational studies were appraised using the Newcastle–Ottawa Scale (NOS) (Wells et al., 2000), while database analysis type studies were assessed using NIH Quality Assessment tool for Observational Cohort and Cross-sectional Studies (National Heart, Lung, and Blood Institute, 2021). Any disagreements in quality assessment were resolved through discussion, with consensus reached among the reviewers prior to final inclusion.

### 2.7 Data synthesis and statistical analysis

Data from included studies were synthesized narratively and quantitatively. Study characteristics, including country, study design, sample size, disease subtype, treatment regimen, and follow-up duration, were summarized descriptively in tables. Clinical efficacy and safety outcomes were also narratively described across studies, including hematologic response, molecular response, symptom improvement, survival outcomes, disease progression, and adverse events.

For quantitative synthesis, proportional meta-analysis was conducted using R software (version 4.5.3) with the meta package (R Core Team, 2024; Schwarzer et al., 2015) for outcomes reported by two or more studies, including complete hematologic response (CHR), partial molecular response (PMR), and grade ≥3 cytopenias. Pooled proportions with 95% confidence intervals were estimated using a generalized linear mixed model with logit transformation under a random-effects framework, with Clopper-Pearson confidence intervals calculated for individual studies. For CHR, subgroup analyses were conducted according to treatment duration (3, 6, 9, 12, 24, and 36 months) to explore temporal trends in response rates. Statistical heterogeneity was assessed using the I^2^ statistic (Higgins et al., 2003), with tau-squared (τ^2^) and Cochran’s Q test additionally reported where applicable. Given the expected small number of studies and heterogeneity in study designs, a formal GRADE assessment of certainty of evidence, publication bias, and sensitivity analyses were not feasible.

## 3 Results

Our initial database search retrieved 507 articles published between 2020 and October 2025 (PUBMED: 92, Web of Science: 182, and Embase: 233). Following removal of 238 duplicates, and title/abstract screening of 269 articles, 91 full-text articles were assessed, of which only 13 met eligibility criteria (Barbui et al., 2021; Gisslinger et al., 2020; Huang et al., 2021; Barbui et al., 2023; Popova-Labachevska et al., 2023; Barbui et al., 2024; Suo et al., 2024; Qin et al., 2024; Palandri et al., 2024; Kirito et al., 2024; Reeves et al., 2025; Yoon et al., 2025; Lee et al., 2025). An additional five highly relevant studies published in between November 2025 and February 2026 were identified via updated search in PUBMED on 11 March 2026 and included in the review after going through the same screening process (Mesa et al., 2025; Chen et al., 2024; Christen et al., 2025; Chang et al., 2025; Inano et al., 2026), resulting in a total of 18 included studies. Case reports/case series were excluded; and in case of extension trials, the original study report was excluded and only the most recent report/extension study’s results were included to avoid duplication. All the 18 studies were used to synthesis qualitative data, while only 11 studies (Barbui et al., 2021; Gisslinger et al., 2020; Mesa et al., 2025; Barbui et al., 2023; Suo et al., 2024; Palandri et al., 2024; Kirito et al., 2024; Yoon et al., 2025; Chen et al., 2024; Christen et al., 2025; Inano et al., 2026) were included in meta-analysis. The selection process is detailed in Figure 1 (PRISMA flow diagram).

**FIGURE 1:**
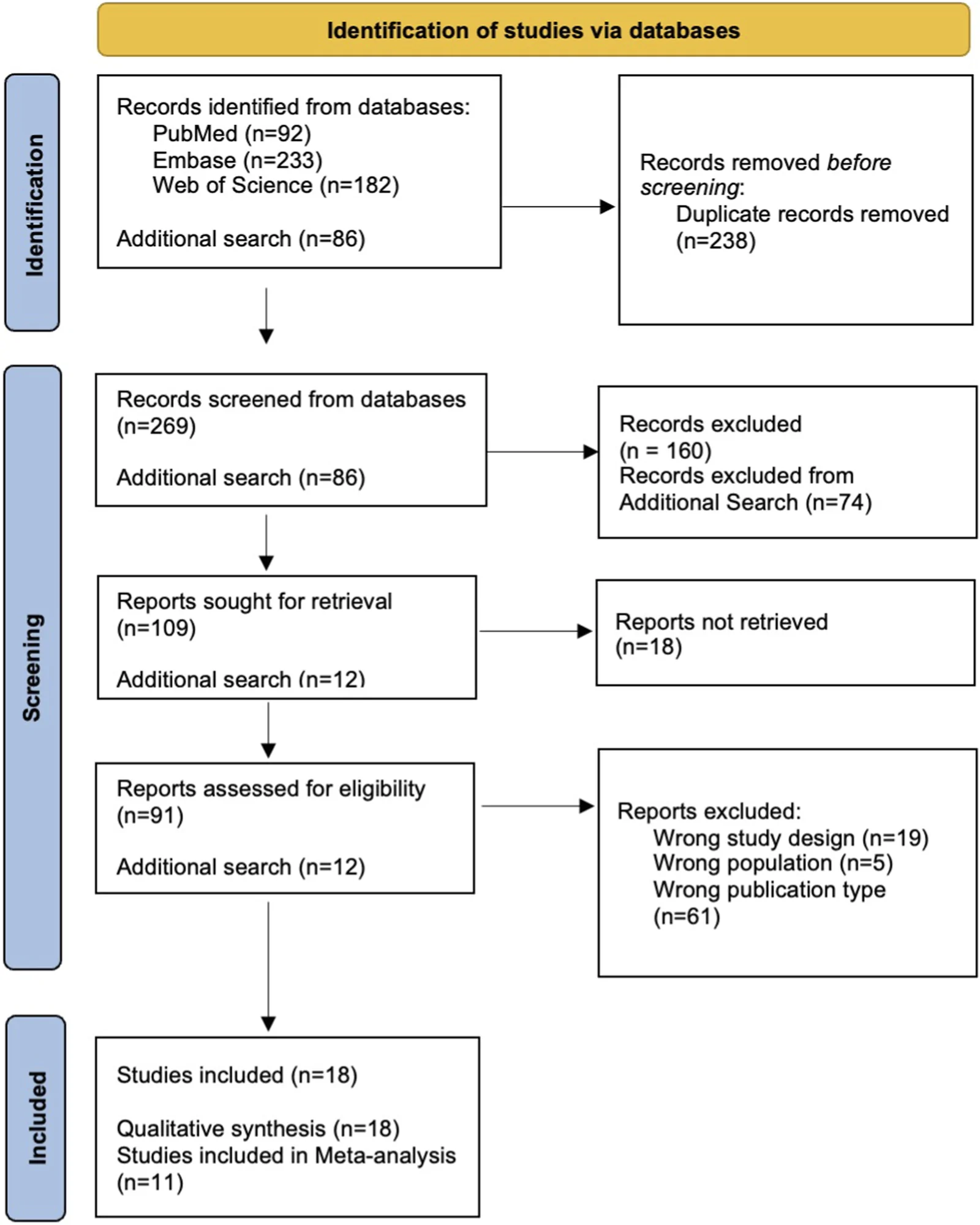
PRISMA flowchart.

### 3.1 Overview of included studies

The included studies comprised five randomized controlled trials (RCTs) (Barbui et al., 2021; Gisslinger et al., 2020; Mesa et al., 2025; Barbui et al., 2023; Barbui et al., 2024), four single arm trials (Suo et al., 2024; Kirito et al., 2024; Yoon et al., 2025; Inano et al., 2026), seven cohorts/observational studies (Huang et al., 2021; Popova-Labachevska et al., 2023; Palandri et al., 2024; Lee et al., 2025; Chen et al., 2024; Christen et al., 2025; Chang et al., 2025), 1 FDA Adverse Event Reporting System (FAERS) database analysis (Reeves et al., 2025), and one secondary analysis of a single arm phase 2 trial (Qin et al., 2024). The included studies were published between 2020 and February 2026 and originated from Asia (n = 10) (Gisslinger et al., 2020; Mesa et al., 2025; Huang et al., 2021; Suo et al., 2024; Qin et al., 2024; Kirito et al., 2024; Yoon et al., 2025; Chen et al., 2024; Chang et al., 2025; Inano et al., 2026), Europe (n = 6) (Barbui et al., 2021; Barbui et al., 2023; Popova-Labachevska et al., 2023; Barbui et al., 2024; Palandri et al., 2024; Christen et al., 2025), and North America (n = 2) (Reeves et al., 2025; Lee et al., 2025) (see Table 1). The pooled sample size across all included studies is 12,785, out of which 1,836 were ropeginterferon users. Several studies focused exclusively on PV (n = 11) (Barbui et al., 2021; Gisslinger et al., 2020; Barbui et al., 2023; Barbui et al., 2024; Suo et al., 2024; Qin et al., 2024; Kirito et al., 2024; Reeves et al., 2025; Yoon et al., 2025; Lee et al., 2025; Inano et al., 2026), while six others included mixed MPN populations consisting of PV, ET, and early or overt MF (Huang et al., 2021; Popova-Labachevska et al., 2023; Palandri et al., 2024; Chen et al., 2024; Christen et al., 2025; Chang et al., 2025), and one study exclusively recruited ET patients (Mesa et al., 2025). About 11 studies were single arm cohorts or trials (Huang et al., 2021; Popova-Labachevska et al., 2023; Suo et al., 2024; Qin et al., 2024; Palandri et al., 2024; Kirito et al., 2024; Yoon et al., 2025; Chen et al., 2024; Christen et al., 2025; Chang et al., 2025; Inano et al., 2026), while the rest had one or many comparator group(s), such as phlebotomy (n = 3) (Barbui et al., 2021; Barbui et al., 2023; Barbui et al., 2024), HU (n = 2) (Gisslinger et al., 2020; Reeves et al., 2025), ruxolitinib (n = 1) (Reeves et al., 2025), peg-interferon (n = 2) (Reeves et al., 2025; Lee et al., 2025), anagrelide (n = 1) (Mesa et al., 2025), or best available therapy (n = 1) (Gisslinger et al., 2020). Follow-up duration varied considerably across studies, with many reporting median follow-up periods ranging from approximately 1 year (Christen et al., 2025) to over 5 years (Chang et al., 2025).

**TABLE 1:** Study characteristics.

Study ID	Country	Study design	Total sample size	No. of participants per arm	Disease subtype	Follow-up duration	Comparator (if any)
Barbui et al. (2021)	Italy	Phase 2 RCT	100	Phlebotomy group (n = 50) Ropeg group (n = 50)	PV	12.1 months (IQR 12.0–12.6)	Phlebotomy + low-dose aspirin
Gisslinger et al. (2020)	Russia	RCT (PROUD-PV) and its extension (CONTINUATION-PV)	PROUD-PV: 257 CONTINUATION-PV: 171	PROUD-PV: • Ropeg group (n = 127) • HU group (n = 127) CONTINUATION-PV: • Ropeg group (n = 95) • BAT group (n = 76)	PV	Ropeg group: 182.1 weeks (166.3–201.7) HU group: 164.5 weeks (144.4–169.3)	Hydroxyurea
Mesa et al. (2025)	Multinational (55 sites across China, Japan, Taiwan, Hong Kong, South Korea, United States, Singapore, Canada)	Open-label RCT phase 3 trial	174	Ropeg group (n = 91) Anagrelide (n = 83)	ET	12.5 months (IQR: 11.5–12.9)	Anagrelide
Huang et al. (2021)	Taiwan	Retrospective observational study	9	Single arm (n = 9)	PV, ET, prePMF, Post-ET MF	54 weeks (26–90)	None
Barbui et al. (2023)	Italy	Phase 2, open label, RCT	127	Ropeg + Phlebotomy (EXP) (n = 64) Phl-O (n = 63)	PV	24 months	Phlebotomy + low-dose aspirin
Popova-Labachevska et al. (2023)	Republic of North Macedonia	Retrospective observational study	16	Single arm (n = 16)	ET, PV, Pre-MF	24 months	NR
Barbui et al. (2024)	Italy	Phase 2 RCT	127	Ropeg arm (n = 64) Phlebotomy arm (n = 63)	PV	Up to 5 years	Phlebotomy
Suo et al. (2024)	China	Phase 2, single-arm study	49	Single arm (n = 45)	PV	NR	None
Qin et al. (2024)	China	Secondary analysis of a single-arm Phase 2 trial	49	Single arm (n = 49)	PV	24 weeks	None
Palandri et al. (2024)	Italy	Retrospective cohort	18	Single arm (n = 18)	PV, ET, prePMF, Post-ET MF	Mean follow-up: Low-risk: 24.8 months (3.3–34.8) High-risk: 18.1 months (4.4–36.1)	None
Kirito et al. (2024)	Japan	Extension of a Phase 2, open-label, single-arm study	27	Single arm (n = 27)	PV	36 months	None
Reeves et al. (2025)	United States	FAERS Database Analysis	11,521	Ropeg (n = 1,008) HU (n = 1,065) peg-IFN (n = 284) Ruxolitinib (n = 9,397)	PV and PV + ET	NR	HU, peginterferon alfa-2a (peg-IFN), Ruxolitinib
Yoon et al. (2025)	South Korea	Phase 2, single-arm study, open-label	95	Single arm (n = 95)	PV	48 weeks	None
Lee et al. (2025)	United States	Retrospective, propensity-matched cohort study	86	Ropeg (n = 43) PEG (n = 43)	PV	Ropeg: 1.8 years PEG: 2 years	Peginterferon alfa-2a (PEG)
Chen et al. (2024)	Taiwan	Retrospective observational study	21	Single arm (n = 21)	PV, ET, Pre-PMF, post-PV/ET MF	NR	None
Christen et al. (2025)	Switzerland	Retrospective observational study	20	Single arm (n = 20)	PV, ET, prePMF, MPN-U	11.5 months (1.9–41.2)	None
Chang et al. (2025)	Taiwan	Retrospective observational study	64	Single cohort (n = 64)	PV, ET, preMF, overt MF	5.3 years	None
Inano et al. (2026)	Japan	Phase 3b, open-label, single-arm, study	21	Single arm (n = 21)	PV	∼24 weeks	None

Abbreviations: NR, not reported; BAT, best available treatment; EXP, experimental; ET, essential thrombocythemia; FAERS, FDA, adverse event reporting system; HU, hydroxyurea; IQR, interquartile range; MF, myelofibrosis; MPN-U, myeloproliferative neoplasm, unclassifiable; PEG, peginterferon alfa-2a; peg-IFN, pegylated interferon; PMF, primary myelofibrosis; PV, polycythemia vera; RCT, randomized controlled trial; Ropeg, ropeginterferon alfa-2b; Phl-O, phlebotomy-only arm.

### 3.2 Population and disease characteristics

Baseline demographic and clinical characteristics varied across studies (see Table 2). The mean/median age ranged from 36 to 64.9 years (Popova-Labachevska et al., 2023; Palandri et al., 2024), and male-to-female ratio was generally balanced, with several cohorts reporting roughly equal numbers of males and females or a slight male predominance. As mentioned earlier, a large proportion of patients were diagnosed with PV, followed by ET, and MF. Across the included studies, disease risk stratification was not uniformly reported and, when available, was based on established systems, including NCCN, ELN, DIPSS, MIPSS70, GIPSS, MYSEC-PM, IPSET, or study-specific PV and ET risk classifications. Seven studies predominantly enrolled patients with low-risk PV (Barbui et al., 2021; Gisslinger et al., 2020; Barbui et al., 2023; Barbui et al., 2024; Yoon et al., 2025; Christen et al., 2025; Inano et al., 2026). Majority of studies (n = 15) included patients who had received some kind of cytoreductive therapy, such as HU, phlebotomy, etc., prior to trial with ropeginterferon (Barbui et al., 2021; Gisslinger et al., 2020; Mesa et al., 2025; Huang et al., 2021; Barbui et al., 2023; Popova-Labachevska et al., 2023; Suo et al., 2024; Palandri et al., 2024; Kirito et al., 2024; Yoon et al., 2025; Lee et al., 2025; Christen et al., 2025; Chang et al., 2025; Inano et al., 2026). Most ropeginterferon studies enrolled predominantly *JAK2* V617F–mutated patients. The prevalence of *JAK2* mutations was consistently high in PV cohorts (90%–100%) (Barbui et al., 2021; Gisslinger et al., 2020; Barbui et al., 2023; Barbui et al., 2024; Suo et al., 2024; Qin et al., 2024; Kirito et al., 2024; Yoon et al., 2025; Christen et al., 2025; Inano et al., 2026), while ET cohorts showed more variable rates (62%–79%) (Mesa et al., 2025; Popova-Labachevska et al., 2023) with *CALR* and *MPL* mutations comprising the remaining minority of cases (Mesa et al., 2025; Popova-Labachevska et al., 2023; Chen et al., 2024; Chang et al., 2025). Baseline *JAK2* V617F allele burden varied widely, with most low-risk PV cohorts reporting medians <50% and higher-risk cohorts generally ≥50% (Barbui et al., 2021; Gisslinger et al., 2020; Mesa et al., 2025; Barbui et al., 2023; Suo et al., 2024; Qin et al., 2024; Kirito et al., 2024; Yoon et al., 2025; Chen et al., 2024; Chang et al., 2025; Inano et al., 2026); however, this pattern was not universal. For instance, Palandri et al., 2024 reported a mean burden of 57% in low-risk patients and 17% in high-risk patients (Palandri et al., 2024), and Christen et al., 2025 reported a median burden of 12% in a mixed MPN cohort (Christen et al., 2025). Where reported, baseline spleen size was modest in low-risk PV and ET (medians around 2–3 cm or 13 cm in PROUD/CONTINUATION-PV and Low-PV) (Barbui et al., 2021; Gisslinger et al., 2020; Mesa et al., 2025; Barbui et al., 2023), but a substantial proportion of pre-PMF/MF patients had splenomegaly at study entry in one study (Chang et al., 2025).

**TABLE 2:** Baseline patient characteristics and treatment details.

Study ID	Age (median, range/IQR or mean ± SD)	Male (n, %)	Prior treatments or first-line or mixed cohort	Molecular status (*JAK2 V617F, CALR, MPL* mutation, allele burden) n, %	Ropeg monotherapy or combination	Ropeg schedule (dose, range, adjustments, weekly/biweekly, etc.)	Duration of therapy (median, range)
Barbui et al. (2021)	Phlebotomy group: 48.1 (IQR: 44.8–57.3) Ropeg group: 51.4 (IQR: 45.4–54.5)	Phlebotomy group: 31 (62%) Ropeg group: 37 (74%)	Mixed	*JAK2* V617F: • Phlebotomy: 41 • Ropeg: 40	Combination (Ropeg + phlebotomy + low-dose aspirin)	Fixed dose of 100 µg SQ every 2 weeks	NR
Gisslinger et al. (2020)	PROUD-PV: • Ropeg group: 60 (range 30–85) • HU group: 60 (range 21–81) CONTINUATION-PV: • Ropeg group: 58 (range 30–85) • BAT group: 59 (range 32–79)	PROUD-PV: • Ropeg group: 59 (46%) • HU group: 60 (47%) CONTINUATION-PV: • Ropeg group: 47 (49%) • BAT group: 36 (47%)	Mixed	*JAK2* V617F: PROUD-PV: • Ropeg (n = 127): 99% (126) • HU (n = 127): 98% (125) CONTINUATION-PV: • Ropeg (n = 95): 99% (94) • BAT (n = 76): 97% (74)	Monotherapy (+ aspirin unless contraindicated)	Starting dose of 100 µg (or 50 µg if transitioning from HU); SQ every 2 weeks	6–36 months
Mesa et al. (2025)	61 years (range: 21–80; IQR: 50–69)	44 (48%)	Received prior treatments	*JAK2* V617F: • Ropeg: 72 (79%) • Anagrelide: 70 (84%) CALR: • Ropeg: 11 (12%) • Anagrelide: 10 (12%) MPL: • Ropeg: 1% • Anagrelide: 1%	Monotherapy	Starting at 250 µg SQ every 2 weeks, with stepwise adjustments to 350 μg and 500 µg	NR
Huang et al. (2021)	50 years (range 30–78)	5 (55.6%)	Received prior treatments	*JAK2* V617F: 8 (88.9%) Triple-negative: 1 (11.1%)	Monotherapy	Starting dose 250 mcg Q2W, escalated by 100 mcg Q2W to target 500 mcg by week 6	54 weeks (26–90)
Barbui et al. (2023)	Phl-O: 48.2 years (IQR 43.7–57.4) EXP: 51.7 years (IQR 45.5–55.3)	Phl-O: 39 (61.9%) EXP: 47 (73.4%)	First line	*JAK2* V617F: 100%	Combination (Ropeg + phlebotomy)	100 µg every 2 weeks	24 months
Popova-Labachevska et al. (2023)	36 years (range 17–51)	5 (31.2%)	Mixed	*JAK2* V617F: 10 (62.5%) *CALR*: 3 (18.8%) Triple-negative: 3 (18.7%)	Monotherapy	Starting dose of 100 mcg with allowed dose escalation of 50 mcg every 2 weeks if needed	NR
Barbui et al. (2024)	NR	NR	First line	Non-Responders: 34%; Responders: 21%^†^	Monotherapy (with low-dose aspirin 100 mg/day)	100 µg every 2 weeks	3.15 years (range 0.04–5.77)
Suo et al. (2024)	Mean: 53.0 years (SD: 10.9)	31 (63.3%)	Received prior treatment	*JAK2* V617F: 100%	Monotherapy	Dosing regimen: 250 μg at Week 0; 350 μg at Week 2; 500 μg at Week 4 (biweekly SQ injection)	1 year
Qin et al. (2024)	56 years (range 29–70)	31 (64.6%)	NR	NR	Monotherapy	250 µg (Week 0), 350 µg (Week 2), 500 µg (Week 4) → maintenance 500 µg every 2 weeks up to 52 weeks	Up to 52 weeks
Palandri et al. (2024)	Low-risk: 47.0 years (range 38.5–58.6) High-risk: 64.9 years (range 52.8–79.3)	13 (72.2%)	Received prior treatments	*JAK2* V617F: 100%	Combination (n = 7) Monotherapy (n = 11)	Starting dose: 100 mcg SQ. Max dose: 200 mcg SQ/2 weeks	Mean time on Ropeg: 15.8 months
Kirito et al. (2024)	Median: 54 years (range: 26–72)	11 (40.7%)	Mixed	*JAK2* V617F: 26 (96.3%)	Monotherapy (concomitant low-dose aspirin allowed)	Mean dose: 396.3 ± 133.0 µg; SQ every 2 weeks	NR
Reeves et al. (2025)	Overall mean age: 67.7 years (SD: 13.0) Ropeg mean age: 59.6 (SD 14.3)	Overall: 1,533 (13.3%) Ropeg: 399 (39.6%)	NR	NR	Monotherapy: 993 reports Combination: 20 reports	NR	NR
Yoon et al. (2025)	58 years (range 25–81)	51 (53.7%)	Mixed	*JAK2* V617F: 100%	Monotherapy	Starting dose: 250 μg at week 1, 350 μg at week 3, 500 μg at week 5, then 500 µg every 2 weeks until week 48	Up to 48 weeks
Lee et al. (2025)	Ropeg: 54 (range 23–78) PEG: 58 (range 32–86)	Ropeg: 21 (49%) PEG: 16 (37%)	Mixed	NR	Monotherapy	Started at 50 or 100 mcg every 2 weeks, uptitrated by 50 mcg if needed	Ropeg: 1.6 years (range 0.1–2.3) PEG: 2 years (range 0.1–2)
Chen et al. (2024)	45.6 years (range 15.7–86.4)	10 (47.6%)	Mixed	*JAK2* V617F: 19 (90.5%) *CALR*: 1 (4.8%) Triple-negative: 1 (4.8%)	Monotherapy	Starting dose 250 µg Q2W, increased by 100–150 µg Q2W to target dose of 500 µg by Week 4 or 6. Average monthly dose was 841 µg in the first year and 624 µg from the second year on	NR
Christen et al. (2025)	52.9 (range 34–77)	11 (55%)	Received prior treatments	*JAK2* V617F: 20 (100%) Additional mutations (e.g., *ASXL1, DNMT3A*): 5/9	Monotherapy	Starting dose mean ± SD: 115.7 ± 78.7 µg Q2W. Maintenance dose mean ± SD: 193.8 ± 124.7 µg Q2W. Dosing was individualized	11.5 months (1.9–41.2)
Chang et al. (2025)	PV: 63.8 (range 15.30–82.2) ET: 56.5 (range 23.9–90.6) preMF: 54.2 (range 46.1–93.0) MF: 62.9 (range 36.0–87.2)	32 (50%)	Mixed	*JAK2*: 56/64 (87.5%) *CALR*: 7/64 (10.9%) *MPL*: 2/64 (3.1%)	Combination (n = 19) Monotherapy (n = 45)	Variable: Slow titration (50 mcg Q2W); Rapid titration (250 mcg initial, escalating to 500 mcg in 2 weeks); Intermediate titration	24.3 months (1.0–78.7)
Inano et al. (2026)	Mean: 57.1	11 (52.4%)	Mixed	*JAK2* V617F: 21 (100%)	Monotherapy (+ aspirin as background)	Three-step dose escalation: Day 1: 250 μg, Week 2: 350 μg, Week 4: 500 μg; administered SQ Q2W. Dose could be reduced for tolerability	∼24 weeks

Abbreviations: NR, not reported; BAT, best available treatment; *CALR*, calreticulin; *ASXL1*, additional sex combs-like one mutation; *DNMT3A*, DNA, methyltransferase 3A mutation; EXP, experimental; HU, hydroxyurea; IQR, interquartile range; *JAK2*, Janus kinase 2; *MPL*, myeloproliferative leukemia virus oncogene mutation; PEG, peginterferon alfa-2a; Phl-O, phlebotomy-only arm; SD, standard deviation; SQ, subcutaneous; Ropeg, ropeginterferon alfa-2b.

† Responders were patients who achieved the primary endpoint of the Low-PV, trial, defined as maintaining a hematocrit <45% throughout the 12-month treatment period without disease progression or the need for additional cytoreductive therapy. Non-responders were patients who did not meet these criteria.

### 3.3 Treatment regimens and follow-up

Sixteen out of the eighteen included studies reported that either the entire cohort or a single study arm received ropeginterferon as monotherapy (Gisslinger et al., 2020; Mesa et al., 2025; Huang et al., 2021; Popova-Labachevska et al., 2023; Barbui et al., 2024; Suo et al., 2024; Qin et al., 2024; Palandri et al., 2024; Kirito et al., 2024; Reeves et al., 2025; Yoon et al., 2025; Lee et al., 2025; Chen et al., 2024; Christen et al., 2025; Chang et al., 2025; Inano et al., 2026), with four of these studies also administering low-dose aspirin as background therapy (Gisslinger et al., 2020; Barbui et al., 2024; Kirito et al., 2024; Inano et al., 2026); Five of eighteen studies included treatment arms in which patients received ropeginterferon in combination with phlebotomy, hydroxyurea (HU), and/or ruxolitinib (Barbui et al., 2021; Barbui et al., 2023; Palandri et al., 2024; Reeves et al., 2025; Chang et al., 2025). Starting doses of ropeginterferon were 100 µg per week or 250 µg every 2 weeks and raised up to a maintenance level of 350 µg or 500 µg every 2 weeks (Barbui et al., 2021; Gisslinger et al., 2020; Mesa et al., 2025; Huang et al., 2021; Barbui et al., 2023; Popova-Labachevska et al., 2023; Barbui et al., 2024; Suo et al., 2024; Qin et al., 2024; Palandri et al., 2024; Yoon et al., 2025; Lee et al., 2025; Chen et al., 2024; Christen et al., 2025; Chang et al., 2025; Inano et al., 2026). Few studies evaluated dose escalation strategies, where treatment was initiated at lower doses and gradually increased based on clinical response and tolerability (n = 4) (Suo et al., 2024; Palandri et al., 2024; Lee et al., 2025; Inano et al., 2026). Treatment duration was variably reported across studies, with median or mean treatment durations ranging from approximately 6 months to 3.15 years (reported ranges, where available, extending from 0.04 to 5.77 years) (Gisslinger et al., 2020; Huang et al., 2021; Barbui et al., 2023; Barbui et al., 2024; Suo et al., 2024; Qin et al., 2024; Palandri et al., 2024; Yoon et al., 2025; Lee et al., 2025; Christen et al., 2025; Chang et al., 2025; Inano et al., 2026).

### 3.4 Efficacy outcomes

#### 3.4.1 Hematologic response

All included studies, except the FAERS database analysis (Reeves et al., 2025), assessed ropeginterferon’s efficacy by evaluating hematological response among patients. Reported response metrics included CHR (n = 12) (Table 3) (Gisslinger et al., 2020; Huang et al., 2021; Popova-Labachevska et al., 2023; Barbui et al., 2024; Suo et al., 2024; Palandri et al., 2024; Kirito et al., 2024; Yoon et al., 2025; Chen et al., 2024; Christen et al., 2025; Chang et al., 2025; Inano et al., 2026), partial hematological response rate (PHR) (n = 2) (Popova-Labachevska et al., 2023; Chang et al., 2025), overall response rate (ORR) (n = 3) (Barbui et al., 2021; Barbui et al., 2023; Barbui et al., 2024), hematocrit control (n = 1) (Barbui et al., 2023), and freedom from phlebotomy in PV cohorts (n = 2) (Barbui et al., 2021; Barbui et al., 2024), all of which reported positive results for ropeginterferon users. Across individual studies, CHR rates varied widely depending on treatment duration and patient population, with generally higher response rates observed with longer exposure to therapy (Gisslinger et al., 2020; Barbui et al., 2024; Suo et al., 2024; Kirito et al., 2024; Yoon et al., 2025; Inano et al., 2026). Median time to achieve CHR ranged from 16 weeks (Huang et al., 2021) to 8.9 months (Palandri et al., 2024).

**TABLE 3:** Efficacy outcomes.

Study ID	Complete hematologic response (CHR) (n, %)	Complete molecular response (CMR) (n, %)	Partial molecular response (n, %)	Mean change in allele burden (baseline vs. follow-up, % ± SD/CI)	Progression-free survival (PFS) (%)	12-Month overall survival (%)	Progression to AML/MF (n, %)
Barbui et al. (2021)	NR	NR	NR	Phlebotomy: Mean change +1.03% (95% CI −2.81 to 4.87) Ropeg: Mean change −10.43% (95% CI −16.97 to −3.89) (p = 0.0060)	Ropeg: 100% Phlebotomy: 84%	NR	0
Gisslinger et al. (2020)	PROUD-PV at month 12: • Ropeg: 53 (43%) • HU: 57 (46%) CONTINUATION-PV at month 36: • Ropeg: 67 (71%) • BAT group: 38 (51%)	PROUD-PV at month 12: • Ropeg: 42 (34%) • HU: 52 (42%) CONTINUATION-PV at month 36: • Ropeg: 62 (66%) • BAT group: 20 (27%)	NR	Ropeg: from 41.9% (SD 23.49) to 30.7% (SD 22.66) HU: from 42.8% (SD 24.14) to 25.9% (SD 21.49)	NR	NR	2 (1.57%)
Mesa et al. (2025)	NR	*JAK2* V617F: • Ropeg: 0 • Anagrelide: 0 *CALR*: • Ropeg: 2 (20%) • Anagrelide: 0	*JAK2* V617F: • Ropeg: 10 (31%) • Anagrelide: 0 *CALR*: • Ropeg: 1 (10%) • Anagrelide: 0	*JAK2* VAF: Ropeg: −21.3% Anagrelide: +12.3%	NR	100% for Ropeg	Ropeg: 0 Anagrelide: 2
Huang et al. (2021)	NR	0%	5 (62.5%)	NR	NR	100%	2 (22.2%)
Barbui et al. (2023)	NR	NR	At 12 months: EXP: 16/55 (29.1%); Phl-O: 0/43 (0%)	At 12 months: EXP: Mean change −11.9% (SD 20.7) Phl-O: Mean change +1.8% (SD 9.0) At 24 months: EXP: Mean change −23.1% (SD 28.9) Phl-O: Mean change +5.4% (SD 30.5)	EXP: 100% Phl-O: 87.3%	NR	0
Popova-Labachevska et al. (2023)	First-line subgroup: 4/7 (57.1%) Previously treated subgroup: 8/9 (88.9%)	NR	NR	NR	100%	NR	0
Barbui et al. (2024)	At year 3: 97% At year 4: 94% At year 5: 94%	NR	NR	NR	Ropeg: 100% Phlebotomy: 87%	NR	1 (1.5%)
Suo et al. (2024)	Week 24: 61.2% Week 36: 69.4% Week 52: 71.4%	2 (4.3%)	By 2009 criteria: 29 (63.0%) By 2013 ELN criteria: 18 (39.1%)	At week 52: −28.4%	NR	100%	0
Qin et al. (2024)	NR	NR	NR	NR	NR	NR	NR
Palandri et al. (2024)	12/18 (66.7%)	NR	3 (16.7%)	NR	100%	100%	0
Kirito et al. (2024)	12 months: 8/27 (29.6%) 24 months: 18/27 (66.7%) 36 months: 22/27 (81.5%)	2 (7.4%)	NR	Median change: −74.8% at 36 months	NR	100%	0
Reeves et al. (2025)	NR	NR	NR	NR	NR	NR	NR
Yoon et al. (2025)	Week 12: 25 (26.6%) Week 24: 40 (46.0%) Week 36: 47 (56.0%) Week 48: 51 (63.0%)	NR	Week 12: 28 (31.8%) Week 24: 29 (35.8%) Week 36: 38 (49.4%) Week 48: 42 (56.8%)	At 48 weeks, by CHR status: CHR responders: Mean reduction of 57.40% ± 26.21 (range: −96.36 to 6.05) CHR non-responders: Mean reduction of 16.14% ± 23.51 (range: −54.27 to 37.37)	NR	100%	1 (1.05%)
Lee et al. (2025)	NR	NR	NR	NR	NR	100%	NR
Chen et al. (2024)	16/20 (80%)	NR	7 (36.8%)	NR	NR	NR	NR
Christen et al. (2025)	12 (60%)	0%	NR	NR	NR	NR	NR
Chang et al. (2025)	36 months: PV 76%; ET 66%; preMF 80%; MF 47%	36 months: PV 22%; ET 13%; preMF 0%; MF 5.6%	NR	Median *JAK2* VAF declined from 67.9% to 18.7% (P < 0.001)	NR	NR	4 (14.3%)
Inano et al. (2026)	Week 12: 5 (23.8%) Week 24: 12 (57.1%)	–	–	Median change from baseline: −8.12%	–	–	–

Abbreviations: NR, not reported; AML, acute myeloid leukemia; MF, myelofibrosis; BAT, best available treatment; EXP, experimental; *CALR*, calreticulin mutation; CHR, complete hematologic response; CI, confidence interval; CMR, complete molecular response; ELN, European LeukemiaNet; ET, essential thrombocythemia; HU, hydroxyurea; Ropeg, ropeginterferon alfa-2b; Phl-O, phlebotomy-only arm; IQR, interquartile range; *JAK2*, Janus kinase 2; PFS, progression-free survival; PV, polycythemia vera; SD, standard deviation; VAF, variant allele frequency.

A total of 13 observations from five studies (Suo et al., 2024; Kirito et al., 2024; Yoon et al., 2025; Chen et al., 2024; Inano et al., 2026) contributed to the proportional meta-analysis of CHR, subgrouped by treatment duration (Figure 1). Individual study proportions ranged from 0.24 to 0.81 across all time points.

At 3 months, the pooled CHR rate was 26% (95% confidence interval (CI): 19%–35%; I^2^ = 0%, τ^2^ = 0, p = 0.79), based on two studies with a combined sample of 115 patients. At 6 months, three studies yielded a pooled CHR of 53% (95% CI: 43%–62%; I^2^ = 36.1%, τ^2^ = 0.0181, p = 0.21), indicating low-to-moderate heterogeneity. At 9 months, two studies demonstrated a pooled CHR of 61% (95% CI: 51%–70%; I^2^ = 56.9%, τ^2^ = 0.0075, p = 0.13). At 12 months, four studies produced a pooled CHR of 59% (95% CI: 43%–74%), with substantial heterogeneity (I^2^ = 76.4%, τ^2^ = 0.3450, p = 0.005). Single-study estimates were available at 24 months (67%; 95% CI: 46%–83%) and 36 months (81%; 95% CI: 62%–94%), both from Kirito et al. (2024), suggesting a continued increase in CHR over time (Kirito et al., 2024).

The overall pooled CHR across all time points and 636 patient-observations was 56% (95% CI: 46%–66%), with high heterogeneity (I^2^ = 81.5%, τ^2^ = 0.4558, p < 0.0001). The test for subgroup differences was statistically significant (χ^2^ = 42.13, df = 5, p < 0.0001), confirming that CHR rates varied significantly across treatment durations, with a trend toward higher response rates with longer treatment exposure (Figure 1).

#### 3.4.2 Molecular response

Molecular response outcomes, including complete molecular response (CMR), partial molecular response (PMR), and changes in *JAK2* allele burden, were variably reported across studies. CMR was uncommonly reported overall. In the PROUD-PV/CONTINUATION-PV trials, CMR occurred in 34% of patients receiving ropeginterferon at 12 months and increased to 66% at 36 months, compared with 42% and 27% in the HU or best available treatment groups, respectively (Gisslinger et al., 2020).

PMR was reported more frequently, with rates ranging from 16.7% (Palandri et al., 2024) to 56.8% (Yoon et al., 2025). Among PV patients, ropeginterferon therapy was consistently associated with reductions in *JAK2* allele burden, with mean decreases of approximately 8.12%–23% within the first 12–24 months (Barbui et al., 2023; Inano et al., 2026) and larger reductions reported with longer follow-up, including median reductions approaching 75% at 36 months (Chang et al., 2025).

Five studies contributed to the pooled analysis of PMR (Figure 2) (Gisslinger et al., 2020; Mesa et al., 2025; Palandri et al., 2024; Yoon et al., 2025; Chen et al., 2024). The pooled PMR rate was 37% (95% CI: 20%–59%), with very high heterogeneity (I^2^ = 90.3%, τ^2^ = 0.8256, p < 0.0001). Notably, the two randomized studies (Gisslinger et al., 2020) and (Mesa et al., 2025) reported divergent results (66% vs. 16%), which may be attributable to differences in comparator arms, dosing regimens, and outcome assessment time points (Figure 3).

**FIGURE 2:**
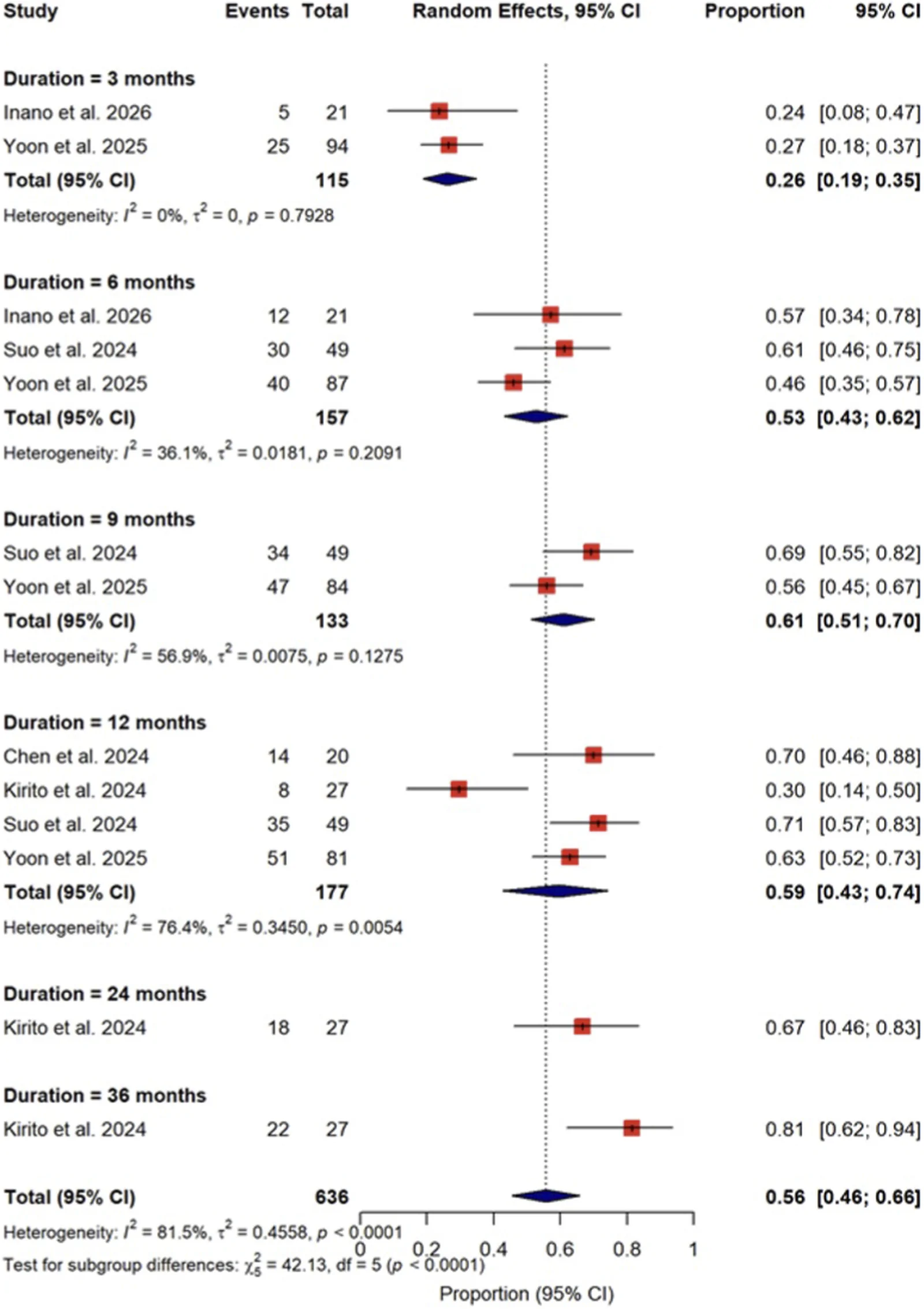
Forest plot of pooled complete hematologic response (CHR) rates with ropeginterferon, subgrouped by treatment duration.

**FIGURE 3:**
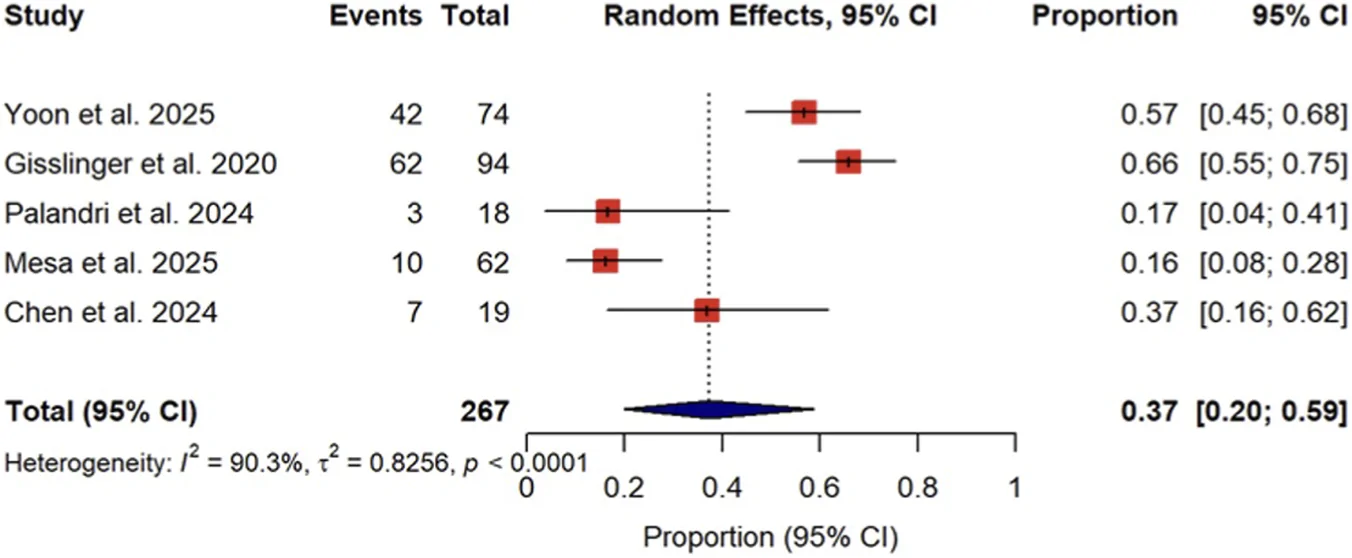
Forest plot of pooled partial molecular response (PMR) rates with ropeginterferon.

#### 3.4.3 Clinical and symptom outcomes

In the PROUD-PV/CONTINUATION-PV trials, the proportion of patients achieving CHR together with normalization of spleen size increased from 21% at 12 months to 53% at 36 months with ropeginterferon, compared with 28% and 38% in the HU or best available treatment groups, respectively (Gisslinger et al., 2020). Additional studies reported reductions in spleen size over time, including mean decreases in spleen area (Barbui et al., 2021; Kirito et al., 2024) and reductions in the proportion of patients with splenomegaly (Barbui et al., 2023).

Symptom improvement was also observed across multiple cohorts. Improvements in MPN-related symptom burden, measured using instruments such as the MPN-SAF Total Symptom Score (TSS), were reported in several studies (n = 7), with six cohorts demonstrating substantial reductions in symptom scores or high rates of symptom control, particularly for pruritus and night sweats (Barbui et al., 2021; Gisslinger et al., 2020; Mesa et al., 2025; Huang et al., 2021; Barbui et al., 2023; Palandri et al., 2024) and one reported statistically insignificant change in MPN TSS (Christen et al., 2025).

#### 3.4.4 Survival and disease progression

Survival outcomes were reported in a limited number of studies. When available, these included OS, PFS, EFS, and DOR. Five studies reported 100% PFS in ropeginterferon users within their respective follow-up periods (Barbui et al., 2021; Barbui et al., 2023; Popova-Labachevska et al., 2023; Barbui et al., 2024; Palandri et al., 2024); and seven studies demonstrated 100% overall survival at 12 months, especially in ropeginterferon cohorts (Mesa et al., 2025; Huang et al., 2021; Suo et al., 2024; Palandri et al., 2024; Kirito et al., 2024; Yoon et al., 2025; Lee et al., 2025).

Among studies reporting progression outcomes (n = 12) (Barbui et al., 2021; Gisslinger et al., 2020; Mesa et al., 2025; Huang et al., 2021; Barbui et al., 2023; Popova-Labachevska et al., 2023; Barbui et al., 2024; Suo et al., 2024; Palandri et al., 2024; Kirito et al., 2024; Yoon et al., 2025; Chang et al., 2025), progression to AML or MF was rare in ropeginterferon-treated patients, with most studies reporting no progression events and only sporadic cases of transformation. The highest progression rate among all of the studies was 4 (14.3%) out of 28 patients with overt MF in the study by Chang et al. (2025).

Thrombosis-free and myelofibrosis-free survival were not explicitly reported as separate time-to-event endpoints in any of the included studies. Most studies reported 0% thrombotic events in ropeginterferon-treated patients, while four studies reported one thrombotic event each (Mesa et al., 2025; Palandri et al., 2024; Lee et al., 2025; Chen et al., 2024). Two studies reported slightly higher numbers: Gisslinger et al. (2020) reported 4 (3%) major thromboembolic events in both the ropeginterferon and hydroxyurea groups, and Chang et al. (2025) reported three arterial thrombotic events, all occurring in patients who had not achieved CHR (Gisslinger et al., 2020; Chang et al., 2025).

### 3.5 Safety outcomes

Safety outcomes were reported in all of the included studies evaluating ropeginterferon in patients with MPNs. Overall, adverse events (AEs) were common but predominantly low grade, with grade ≥3 toxicities occurring infrequently, often only affecting 1%–2% of the cohorts, except in Kirito et al. (2024) study where the number of patients experiencing grade ≥3 adverse event was 7/27 (25.9%) (Kirito et al., 2024).

#### 3.5.1 Grade ≥ 3 toxicities

Several studies reported no severe cytopenic events, while others observed isolated cases of anemia, neutropenia, or thrombocytopenia. When reported, the incidence of severe hematologic toxicities was generally lower or comparable to that observed with comparator therapies; however, a higher frequency of grade ≥3 neutropenia was reported in the ropeginterferon arm when compared to phlebotomy (average of six patients in ropeginterferon group vs. 0 patients in phlebotomy group) (Barbui et al., 2021; Barbui et al., 2023). In randomized and observational cohorts, hematologic toxicities were typically reversible and manageable with dose modification or temporary treatment interruption. Six studies reported data on grade ≥3 cytopenias associated with ropeginterferon treatment (Figure 4) (Barbui et al., 2021; Barbui et al., 2023; Palandri et al., 2024; Yoon et al., 2025; Christen et al., 2025; Inano et al., 2026). Three of the six studies (Palandri et al., 2024; Christen et al., 2025; Inano et al., 2026) reported zero events, while the remaining studies reported low event rates. The pooled proportion of grade ≥3 cytopenias was 3% (95% CI: 1%–10%), with negligible heterogeneity (I^2^ = 0.0%, τ^2^ = 0.9045, p = 0.49). These findings further indicate that severe cytopenias are an infrequent adverse event with ropeginterferon, occurring in a small minority of treated patients across diverse clinical settings.

**FIGURE 4:**
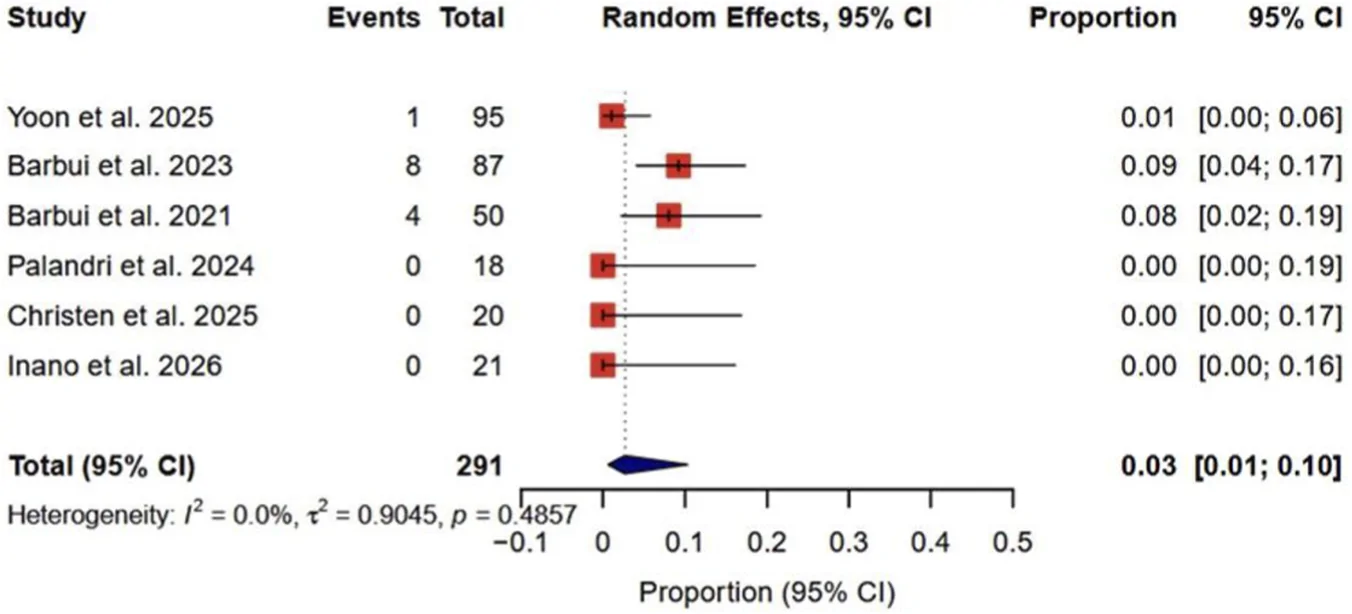
Forest plot of pooled grade ≥3 cytopenias rates with ropeginterferon.

Grade ≥3 hepatic adverse events were reported in most studies but occurred infrequently, generally affecting ≤4–5% of patients receiving ropeginterferon. In the PROUD-PV trial, grade ≥3 alanine aminotransferase (ALT) and aspartate aminotransferase (AST) elevations occurred in 5/127 (4%) and 3/127 (2%) patients receiving ropeginterferon, with no comparable events in the HU arm, while other cohorts reported similarly low rates such as 2/87 (2.3%) hypertransaminasemia and 1%–4.8% transaminitis (Barbui et al., 2021; Gisslinger et al., 2020; Mesa et al., 2025; Huang et al., 2021; Barbui et al., 2023; Barbui et al., 2024; Suo et al., 2024; Qin et al., 2024; Kirito et al., 2024; Yoon et al., 2025; Chen et al., 2024; Chang et al., 2025). Grade ≥3 flu-like symptoms were rare, with most studies reporting 0% incidence (Barbui et al., 2021; Mesa et al., 2025; Huang et al., 2021; Barbui et al., 2023; Palandri et al., 2024; Chen et al., 2024; Christen et al., 2025; Chang et al., 2025; Inano et al., 2026), and occasional cases, such as pyrexia in 2/27 patients (7.4%) reported in a small cohort (Kirito et al., 2024).

#### 3.5.2 Other ≤ grade 2 adverse events and discontinuations

Across the included studies, the most frequently reported adverse events in this column were ALT/AST elevations or transaminitis, followed by increases in β2-microglobulin in urine, gamma-glutamyl transferase (GGT) elevations, and hyperuricemia. Fatigue or asthenia, decreased or loss of weight, hypertriglyceridemia, alopecia, pruritus or itching, and general constitutional symptoms such as malaise and flu-like syndromes appeared next in frequency, with gastrointestinal complaints (diarrhea, reduced appetite), musculoskeletal pain (arthralgia, myalgia, bone pain), anemia or other cytopenias, thyroid dysfunction (including hypothyroidism), skin rash or other skin symptoms, headache or migraine, psychiatric manifestations (depression, mood disturbance, bipolar disorder, insomnia), urinary tract infection, neuropathy or other neurological complaints, and eye disorders being less common (Barbui et al., 2021; Gisslinger et al., 2020; Mesa et al., 2025; Huang et al., 2021; Barbui et al., 2023; Popova-Labachevska et al., 2023; Barbui et al., 2024; Suo et al., 2024; Qin et al., 2024; Palandri et al., 2024; Kirito et al., 2024; Reeves et al., 2025; Yoon et al., 2025; Lee et al., 2025; Chen et al., 2024; Christen et al., 2025; Chang et al., 2025; Inano et al., 2026). All 18 studies reported no treatment-related deaths among ropeginterferon users. Treatment discontinuation rate ranged from 0% (Palandri et al., 2024; Inano et al., 2026) to 32.8% (21/64 patients) in Barbui et al. (2024). The reasons for discontinuations included drug-related toxicity (n = 41 patients), disease progression (n = 3 patients), lack of response (n = 20 patients), and reasons unrelated to drug (n = 41 patients) in all of the ropeginterferon recipients included in this review; these figures are presented as a descriptive summary only; formal pooling was not performed due to heterogeneity in study designs, sample sizes, and follow-up durations across studies (Barbui et al., 2021; Gisslinger et al., 2020; Mesa et al., 2025; Huang et al., 2021; Barbui et al., 2023; Suo et al., 2024; Kirito et al., 2024; Yoon et al., 2025; Chen et al., 2024; Christen et al., 2025; Chang et al., 2025). Notably, pharmacovigilance data, as reported in the FAERS database analysis, shows that across 2022–2024, ropeginterferon did not demonstrate reporting odds ratios (RORs) above one for serious AEs (SAEs) and for fatal AEs (FAEs), with SAEs RORs of 0.28, 0.27, and 0.68, for each year respectively (Reeves et al., 2025). In contrast, ropeginterferon had elevated RORs for non-serious adverse events (5.56 in 2022, 4.34 in 2023, 1.79 in 2024), consistent with a disproportionate reporting of non-serious events that appeared to decrease over time, and an age-stratified analysis showed an increased overall ROR in patients younger than 60 years (2.72, 95% CI 1.19–6.22) but not in those aged 60 years or older (Reeves et al., 2025). Detailed adverse event data for each individual study are presented in Supplementary Table S6.

### 3.6 Risk of bias assessment

The risk was generally low-to-moderate among the randomized trials: all five PV trials (Barbui et al., 2021; Gisslinger et al., 2020; Mesa et al., 2025; Barbui et al., 2023; Barbui et al., 2024) were judged at low risk in the randomization, missing data, and outcome measurement domains, but most had some concerns for deviations from intended interventions or selective reporting, and one ET trial (SURPASS-ET) was rated overall high risk due to issues in adherence and outcome selection (Mesa et al., 2025). Non-randomized interventional studies frequently showed serious or critical risk of bias, driven mainly by confounding and selection of participants (Suo et al., 2024; Qin et al., 2024; Kirito et al., 2024; Yoon et al., 2025; Inano et al., 2026), whereas cohort and real-world studies achieved between five and nine stars on the Newcastle–Ottawa Scale (Huang et al., 2021; Popova-Labachevska et al., 2023; Palandri et al., 2024; Lee et al., 2025; Chen et al., 2024; Christen et al., 2025; Chang et al., 2025), indicating moderate quality overall with Lee et al., 2025 scoring highest and several single-centre series scoring lower due to limited representativeness and adjustment (Lee et al., 2025). The FAERS database (Reeves et al., 2025) analysis’s quality rating was determined to be ‘Fair’. The complete Risk of Bias assessment of all included studies can be found in the Supplementary Material.

## 4 Discussion

This study supports the use of ropeginterferon as an effective and well-tolerated treatment for MPNs. The overall CHR rate across pooled data was 56%. The response substantially increased over time, reaching up to 81% at extended follow-up trials (Kirito et al., 2024). These results are in line with the gradual character of interferon-mediated disease management, in which longer exposure eventually results in more pronounced hematologic reactions. Data from individual trials support this time-dependent impact. In the PROUD-PV/CONTINUATION-PV trial, the proportion of patients achieving CHR with normal spleen size increased from 21% at 12 months to 53% at 36 months, demonstrating progressive improvement with continued treatment, suggesting that optimal clinical benefit is not attained early but rather builds up with continued therapy (Gisslinger et al., 2020). In a similar vein, Kirito et al. (2024) demonstrated that CHR increased with longer follow-up, surpassing 70% in long-term cohorts (Kirito et al., 2024).

The general trend of molecular responses was comparable. While comparative arms (HU/best available therapy) demonstrated diminishing or poorer molecular responses as time passed (42%–27%), CMR in PROUD-PV improved from 34% at 12 months to 66% at 36 months (Gisslinger et al., 2020). PMR was achieved in 37% of patients (95% CI: 20%–59%); however, interpretation of this pooled estimate is limited by differences in response definitions, patient populations, and assessment time points across studies. Nevertheless, consistent reductions in JAK2 allele burden, ranging from approximately 8%–23% within 12–24 months and approaching 75% after 36 months of follow-up, support the disease-modifying potential of ropeginterferon (Barbui et al., 2023; Chang et al., 2025). As molecular response assessment becomes more standardized, quantitative monitoring of driver mutation burden may play an increasingly important role in treatment stratification and long-term disease monitoring, analogous to molecular monitoring in chronic myeloid leukemia. Overall, these efficacy findings were accompanied by a favourable safety profile. From a safety standpoint, ropeginterferon showed a low incidence of major toxicity, with a pooled grade ≥3 cytopenias of 3% (95% CI: 1%–10%) and hepatotoxicity generally ≤4–5% across studies. Most importantly, the majority of adverse events were low-grade and reversible, and no treatment-related mortality was observed in any of the 18 included studies. Although most included studies did not formally evaluate thrombosis-free or myelofibrosis-free survival, the consistently low incidence of thrombotic events and progression to MF across studies suggests that ropeginterferon may confer durable clinical benefit beyond hematologic and molecular responses.

The results of this meta-analysis are in line with previous research assessing interferon-based treatments in MPNs, which have historically shown effectiveness in producing hematologic and molecular responses, especially in ET and PV. In addition, traditional pegylated interferon alfa formulations have demonstrated significant hematologic and molecular activity in PV and ET, including stable responses in longer-term follow-up cohorts and high hematologic response rates in early phase trials. However, tolerability and treatment withdrawal remained major drawbacks with earlier interferon formulations (Daunov and Klisovic, 2025; Quintás-Cardama et al., 2006; Brkic and Meyer, 2021; Kiladjian et al., 2011). Hence, the increasing improvement in response rates seen in this analysis could be explained by ropeginterferon’s longer duration of action and tolerability when compared to previous interferon preparations, as it allows for less frequent administration (Vachhani et al., 2024; Bukowski et al., 2002). Depending on the type of pegylated interferon, its half-life can range from 54 to 168 h, with the third-generation ropeginterferon alfa-2b-njft (BESREMi®) having the longest half-life of 168 h (Vachhani et al., 2024; Dummer and Mangana, 2009).

A previous meta-analysis by Gu et al. (2021) assessed interferon therapy in PV and ET and found that patient age and interferon formulation had an impact on the pooled overall hematologic response (OHR) rate of 86% and molecular response rate of 48% (Gu et al., 2021). This meta-analysis, on the other hand, focuses on ropeginterferon alfa-2b and indicates a lower pooled CHR of 56%; hence, endpoint definition and study composition account for the majority of this discrepancy. Furthermore, our analysis concentrated on the stricter CHR, while the prior meta-analysis employed OHR, which encompasses both complete and partial responses across heterogeneous interferon preparations, including pegylated and conventional interferon. Another recent meta-analysis by Nada et al. (2025) evaluated ropeginterferon alfa-2b exclusively in PV, including eight studies (761 patients), whereas our review provides a broader synthesis across the MPN spectrum, analyzing 18 studies involving 12,785 patients (Nada et al., 2025). Both reviews demonstrate a rise in CHR and converge at three and 12 months (26% vs. 32%, 59% vs. 63%), providing mutual validation, while our review extends to 24 and 36 months. Unlike the previous review, which focused on all-grade adverse events, we quantified clinically significant grade ≥3 toxicities, demonstrating that serious hepatic events and cytopenia were uncommon. Together with pharmacovigilance data and comprehensive quality assessment, this review provides a long-term efficacy and safety evaluation of ropeginterferon across the MPN spectrum.

### 4.1 Clinical implications and future research

The findings of this systematic review and meta-analysis carry significant implications for MPNs management. First, ropeginterferon may be regarded as a first-line cytoreductive treatment for PV, especially in younger individuals or those whose disease modification is demanded (Marchetti et al., 2022; De Fazio et al., 2026; Barbui et al., 2018b). Its application beyond simple cytoreduction is supported by its ability to produce molecular responses, reduce phlebotomy requirements, and provide prolonged hematologic control (Tefferi and Barbui, 2023; Tremblay et al., 2025). Second, new research indicates that ropeginterferon is a useful substitute for traditional treatments such as anagrelide or HU in ET, especially in individuals who are intolerant to or resistant to first-line therapies. This is reinforced by SURPASS-ET and subsequent ET treatment updates, which continue to recognize interferon as a significant therapeutic choice in specific patients, such as those who are younger in age, carry low to intermediate risk of disease, resistant to first-line treatment, and for women who intend to become pregnant or are in the childbearing age, when it is best to avoid leukemogenic or teratogenic medications (Mesa et al., 2025; Barbui et al., 2018b). Third, treatment responses to ropeginterferon are less consistent in MF. Although several included studies enrolled patients with early or prefibrotic MF within mixed MPN cohorts, robust pooled estimates were not feasible due to inadequate and inconsistent reporting of MF-specific outcomes. For instance, hematologic responses varied significantly by subtype in Chang et al. (2025); CHR was found in 80% of preMF (4/5) compared to 42.8% of overt MF (12/28), and overt MF had poorer cumulative response rates over time. While 14.3% of patients with overt MF progressed and those with high-molecular-risk mutations showed no response, molecular responses varied likewise, with CMR rates ranging from 0% to 5.6% and PMR + CMR rates from 42% to 75%. Further restricting interpretability were other included studies (Huang et al., 2021; Palandri et al., 2024; Chen et al., 2024; Christen et al., 2025) that either reported very few MF patients or did not describe MF-specific outcomes. Furthermore, the majority of the information that is currently available indicates that interferon-based treatments are more effective in the early stages of the disease, while their effectiveness in overt or advanced MF is still less certain. On the other hand, JAK inhibitors, and particularly ruxolitinib, remain the standard of therapy in intermediate-to high-risk MF because of their proven effectiveness in reducing spleen volume and controlling symptoms (Harrison et al., 2012; Butler et al., 2025), even though they are not curative (Butler et al., 2025), since the only curative treatment for MF is allogeneic hematopoietic stem cell (Stuckey et al., 2025). Therefore, the benefits of ropeginterferon seen in PV and ET cannot be extrapolated to advanced MF populations based on the available data.

Future research should aim to address these gaps. Studies should further define the optimal positioning of ropeginterferon across the MPN disease spectrum. While hydroxyurea remains an appropriate comparator in PV and ET, dedicated studies in MF should focus on disease stage-specific treatment strategies. Given the complementary mechanisms of interferon and JAK inhibition, evaluation of combination approaches, such as those currently being explored in studies like the FEDORA, may provide greater clinical benefit than direct comparisons alone, particularly in earlier-stage MF (McIlroy et al., 2025). In addition, molecular response criteria and reporting should be standardised to improve study comparability and strengthen future meta-analyses. Further investigation of predictive biomarkers, including baseline allele burden and mutational profile, may help identify patients most likely to benefit from therapy. Long-term outcomes, including progression to MF or acute myeloid leukemia, should also be rigorously evaluated to better define the disease-modifying potential of ropeginterferon. Finally, longer-term real-world studies are needed to further establish its long-term safety profile, durability of response, and effectiveness in routine clinical practice.

### 4.2 Strengths and limitations

This study has several noteworthy strengths. First, it is a thorough and current synthesis of ropeginterferon information that was published between 2020 and 2026, including both real-world data and randomised controlled trials. Second, the results are more generalizable because 18 studies with a variety of patient demographics were included. Third, considering the more gradual yet cumulative impacts of interferon-based therapies, the application of time-stratified meta-analysis offers novel insight into the kinetics of treatment response. Lastly, the general validity and reliability of the results are strengthened by the use of strong statistical methods, such as random-effects modelling and standardized risk-of-bias evaluation. However, a number of limitations should be considered. First, there was significant heterogeneity (I^2^ up to 81.5%), which was caused by variations in the study design, patient demographics (including PV, ET, and MF), dosage approaches, and definitions of outcomes. Second, it is difficult to make definitive comparisons because many of the included studies were observational or single arm. Third, even with subgroup and time-stratified analyses, direct comparisons between studies are complicated by differences in follow-up length and evaluation timepoints. Fourth, variation in pooled PMR estimates was caused by inconsistent definitions and reporting of molecular responses.

## 5 Conclusion

This systematic review suggests that ropeginterferon alfa-2b is an effective and well-tolerated treatment option for Myeloproliferative neoplasms, particularly polycythemia vera. Across the included studies, ropeginterferon use was associated with progressively increasing hematologic and molecular response rates over time, particularly sustained reductions in JAK2 allele burden, supporting its disease-modifying potential. Most patients were also reported of having positive improvements in clinical outcomes and symptoms. The safety profile was favorable, with most adverse events being low grade or manageable, and the severe toxicities, such as grade ≥3 cytopenias and hepatotoxicity, occurring only infrequently. These results support ropeginterferon’s efficacy and tolerability profile as a treatment for MPNs, and highlight its long-term disease-modifying potential, especially in polycythemia vera and essential thrombocythemia.

## Data Availability

The original contributions presented in the study are included in the article/Supplementary Material, further inquiries can be directed to the corresponding author.

## Author’s note

On 31 August 2026, subsequent to acceptance of this manuscript, the FDA approved ropeginterferon alfa-2b-njft (Besremi) for adults with essential thrombocythemia, based on the SURPASS-ET trial discussed herein (Mesa et al., 2025), making it the first FDA-approved therapy specifically for ET in nearly three decades (Food and Drug Administration, 2026).
