# Supplementary material for: Efficacy and safety of ropeginterferon in myeloproliferative neoplasms: a systematic review and meta-analysis
Source: Front Pharmacol. 2026 Sep 23;17:1841557. doi: 10.3389/fphar.2026.1841557 (PMC13643069; doi:10.3389/fphar.2026.1841557)
Supplement: Supplementary file 1 [file Table1.docx]

**Supplementary Material**

# **PRISMA Checklist**

# Supplementary Table S1. PRISMA Checklist.

| **Section and Topic** | **Item #** | **Checklist item** | **Section where item is reported** |
| --- | --- | --- | --- |
| **TITLE** | | |  |
| Title | 1 | Identify the report as a systematic review. | Title page |
| **ABSTRACT** | | |  |
| Abstract | 2 | See the PRISMA 2020 for Abstracts checklist. | Abstract |
| **INTRODUCTION** | | |  |
| Rationale | 3 | Describe the rationale for the review in the context of existing knowledge. | 1. Introduction |
| Objectives | 4 | Provide an explicit statement of the objective(s) or question(s) the review addresses. | 1. Introduction |
| **METHODS** | | |  |
| Eligibility criteria | 5 | Specify the inclusion and exclusion criteria for the review and how studies were grouped for the syntheses. | 2.2 Eligibility criteria |
| Information sources | 6 | Specify all databases, registers, websites, organisations, reference lists and other sources searched or consulted to identify studies. Specify the date when each source was last searched or consulted. | 2.4 Search Strategy and Study Selection |
| Search strategy | 7 | Present the full search strategies for all databases, registers and websites, including any filters and limits used. | 2.2 Eligibility criteria and Supplementary file |
| Selection process | 8 | Specify the methods used to decide whether a study met the inclusion criteria of the review, including how many reviewers screened each record and each report retrieved, whether they worked independently, and if applicable, details of automation tools used in the process. | 2.4 Search Strategy and Study Selection |
| Data collection process | 9 | Specify the methods used to collect data from reports, including how many reviewers collected data from each report, whether they worked independently, any processes for obtaining or confirming data from study investigators, and if applicable, details of automation tools used in the process. | 2.5 Data extraction |
| Data items | 10a | List and define all outcomes for which data were sought. Specify whether all results that were compatible with each outcome domain in each study were sought (e.g. for all measures, time points, analyses), and if not, the methods used to decide which results to collect. | 2.5 Data extraction |
|  | 10b | List and define all other variables for which data were sought (e.g. participant and intervention characteristics, funding sources). Describe any assumptions made about any missing or unclear information. | 2.5 Data extraction |
| Study risk of bias assessment | 11 | Specify the methods used to assess risk of bias in the included studies, including details of the tool(s) used, how many reviewers assessed each study and whether they worked independently, and if applicable, details of automation tools used in the process. | 2.6 Risk of Bias Assessment |
| Effect measures | 12 | Specify for each outcome the effect measure(s) (e.g. risk ratio, mean difference) used in the synthesis or presentation of results. | 2.7 Data Synthesis and Statistical Analysis |
| Synthesis methods | 13a | Describe the processes used to decide which studies were eligible for each synthesis (e.g. tabulating the study intervention characteristics and comparing against the planned groups for each synthesis (item #5)). | 2. Methods |
|  | 13b | Describe any methods required to prepare the data for presentation or synthesis, such as handling of missing summary statistics, or data conversions. | 2.7 Data Synthesis and Statistical Analysis |
|  | 13c | Describe any methods used to tabulate or visually display results of individual studies and syntheses. | 2.7 Data Synthesis and Statistical Analysis |
|  | 13d | Describe any methods used to synthesize results and provide a rationale for the choice(s). If meta-analysis was performed, describe the model(s), method(s) to identify the presence and extent of statistical heterogeneity, and software package(s) used. | 2.7 Data Synthesis and Statistical Analysis |
|  | 13e | Describe any methods used to explore possible causes of heterogeneity among study results (e.g. subgroup analysis, meta-regression). | 2.7 Data Synthesis and Statistical Analysis |
|  | 13f | Describe any sensitivity analyses conducted to assess robustness of the synthesized results. | 2.7 Data Synthesis and Statistical Analysis |
| Reporting bias assessment | 14 | Describe any methods used to assess risk of bias due to missing results in a synthesis (arising from reporting biases). | 2.6 Risk of Bias Assessment |
| Certainty assessment | 15 | Describe any methods used to assess certainty (or confidence) in the body of evidence for an outcome. | 2.7 Data Synthesis and Statistical Analysis |
| **RESULTS** | | |  |
| Study selection | 16a | Describe the results of the search and selection process, from the number of records identified in the search to the number of studies included in the review, ideally using a flow diagram. | 3. Results |
|  | 16b | Cite studies that might appear to meet the inclusion criteria, but which were excluded, and explain why they were excluded. | NA |
| Study characteristics | 17 | Cite each included study and present its characteristics. | Table 1 |
| Risk of bias in studies | 18 | Present assessments of risk of bias for each included study. | 3.6 Risk of Bias Assessment |
| Results of individual studies | 19 | For all outcomes, present, for each study: (a) summary statistics for each group (where appropriate) and (b) an effect estimate and its precision (e.g. confidence/credible interval), ideally using structured tables or plots. | Tables 1-3 |
| Results of syntheses | 20a | For each synthesis, briefly summarise the characteristics and risk of bias among contributing studies. | 3.6 Risk of Bias Assessment |
|  | 20b | Present results of all statistical syntheses conducted. If meta-analysis was done, present for each the summary estimate and its precision (e.g. confidence/credible interval) and measures of statistical heterogeneity. If comparing groups, describe the direction of the effect. | 3.4.1 Hematologic Response  And 3.5.1 Grade ≥3 Toxicities |
|  | 20c | Present results of all investigations of possible causes of heterogeneity among study results. | 3. Results |
|  | 20d | Present results of all sensitivity analyses conducted to assess the robustness of the synthesized results. | 3. Results |
| Reporting biases | 21 | Present assessments of risk of bias due to missing results (arising from reporting biases) for each synthesis assessed. | 3. Results |
| Certainty of evidence | 22 | Present assessments of certainty (or confidence) in the body of evidence for each outcome assessed. | NA |
| **DISCUSSION** | | |  |
| Discussion | 23a | Provide a general interpretation of the results in the context of other evidence. | 4. Discussion |
|  | 23b | Discuss any limitations of the evidence included in the review. | 4. Discussion |
|  | 23c | Discuss any limitations of the review processes used. | 4. Discussion |
|  | 23d | Discuss implications of the results for practice, policy, and future research. | 4. Discussion |
| **OTHER INFORMATION** | | |  |
| Registration and protocol | 24a | Provide registration information for the review, including register name and registration number, or state that the review was not registered. | 2.1 Study Design and Protocol Registration |
|  | 24b | Indicate where the review protocol can be accessed, or state that a protocol was not prepared. | 2.1 Study Design and Protocol Registration |
|  | 24c | Describe and explain any amendments to information provided at registration or in the protocol. | NA |
| Support | 25 | Describe sources of financial or non-financial support for the review, and the role of the funders or sponsors in the review. | 6. Funding |
| Competing interests | 26 | Declare any competing interests of review authors. | 7. Conflict of interest |
| Availability of data, code and other materials | 27 | Report which of the following are publicly available and where they can be found: template data collection forms; data extracted from included studies; data used for all analyses; analytic code; any other materials used in the review. | 8. Data availability |

*Abbreviations: NA, Not applicable*

*From:* Page MJ, McKenzie JE, Bossuyt PM, Boutron I, Hoffmann TC, Mulrow CD, et al. The PRISMA 2020 statement: an updated guideline for reporting systematic reviews. BMJ 2021;372:n71. doi: 10.1136/bmj.n71. This work is licensed under CC BY 4.0. To view a copy of this license, visit <https://creativecommons.org/licenses/by/4.0/>

# **Search Strategy**

Search done on: 17 October 2025

PUBMED: 92 Articles

(("Myeloproliferative Disorders"[MeSH Terms] OR "Myeloproliferative Neoplasm"[Title/Abstract] OR

"Myeloproliferative Neoplasms"[Title/Abstract] OR "MPN"[Title/Abstract] OR

"myeloproliferative*"[Title/Abstract] OR "Polycythemia"[Title/Abstract] OR "Polycythemia

Vera"[MeSH Terms] OR "Essential Thrombocythemia"[Title/Abstract] OR "essential

thrombocytosis"[Title/Abstract] OR "Myelofibrosis"[Title/Abstract]) AND ("peginterferon alfa-

2b"[Supplementary Concept] OR "peginterferon alfa-2b"[Title/Abstract] OR

"ropeginterferon"[Title/Abstract] OR "ropeg"[Title/Abstract]) AND 2020/01/01:2025/12/31[Date -

Publication]) AND (english[Filter])

Web of Science: 182 Articles

(((((((ALL=(Myeloproliferative Disorder)) OR ALL=(Myeloproliferative Neoplasm*)) OR

ALL=(myeloproliferative* )) OR ALL=(Polycythemia Vera)) OR ALL=(Essential Thrombocythemia)) OR

ALL=(Essential Thrombocytosis)) AND ALL=(ropeginterferon)) OR ALL=(ropeg*) and 2024 or 2025 or

2023 or 2022 or 2021 or 2020 (Publication Years) and English (Languages)

EMBASE: 233 Articles

('myeloproliferative disorder'/exp OR 'polycythemia vera'/exp OR 'thrombocythemia'/exp OR

'myelofibrosis'/exp OR 'polycythemia vera':ti,ab OR 'essential thrombocythemia':ti,ab OR

'myelofibrosis':ti,ab) AND ('ropeginterferon alpha2b'/exp OR ropeginterferon:ti,ab OR ropeg:ti,ab)

AND [2020-2025]/py AND [english]/lim

# **Assessments of the Quality of Included Studies:**

**Supplementary Table S2 Quality Assessment using RoB2**

Sterne JAC, Savović J, Page MJ, Elbers RG, Blencowe NS, Boutron I, et al. RoB 2: a revised tool for assessing risk of bias in randomised trials. *BMJ*. 2019;366:l4898.

<https://www.riskofbias.info/welcome/rob-2-0-tool/current-version-of-rob-2>

| Author & year | Domain 1 | Domain 2 | Domain 3 | Domain 4 | Domain 5 | Overall Risk of Bias |
| --- | --- | --- | --- | --- | --- | --- |
| Barbui et al., 2024 | Low | Some concerns | Some concerns | Low risk | Low | Some concerns |
| Barbui et al., 2023 | Low | Low | Low | S Some concerns | Low | Some concerns |
| Gisslinger, H 2020 | Low | Some Concerns | Low | Low | Some concerns | Some Concerns |
| Barbui, T 2021 | Low | Some Concerns | Low | Low | Some concerns | Some concerns |
| Mesa, R 2025 | Low | Some Concerns | High risk | High risk | Low | High |

**Supplementary Table S3 Quality Assessment using ROBINS-I**

Sterne JA, Hernán MA, Reeves BC, Savović J, Berkman ND, Viswanathan M, et al. **ROBINS-I: a tool for assessing risk of bias in non-randomised studies of interventions. *BMJ*. 2016;355:i4919.**

<https://www.riskofbias.info/welcome/robins-i-v2>

| Author & year | Domain 1 | Domain 2 | Domain 3 | Domain 4 | Domain 5 | Domain 6 | Domain 7 | Overall Risk of Bias |
| --- | --- | --- | --- | --- | --- | --- | --- | --- |
| Suo et al., 2024 | Serious | Low | Low | Moderate | Low | Low | Moderate | Serious |
| Qin et al., 2024 | Serious | Low | Low | Moderate | Moderate | Low | Moderate | Serious |
| Yoon et al., 2025 | Serious | Low | Low | Moderate | Moderate | Low | Moderate | Serious |
| Kirito, K 2024 | Critical | Moderate | Critical | Low | Moderate | Low | Low | Critical |
| Inano, T 2026 | Critical | Low | Low | Low | Low | Low | Moderate | Critical |

**Supplementary Table S4 Quality Assessment using New Castle Ottawa Scale for Case Control and Cohort Studies**

Wells GA, Shea B, O’Connell D, Peterson J, Welch V, Losos M, et al. The Newcastle-Ottawa Scale (NOS) for assessing the quality of nonrandomised studies in meta-analyses.

<https://ohri.ca/en/who-we-are/core-facilities-and-platforms/ottawa-methods-centre/newcastle-ottawa-scale>

| Author & Year | Representativeness of the cohort | Selection of the non-exposed cohort | Ascertainment of exposure | Outcome of interest was not present at start | Controls for most important factor | Controls other relevant factors | Assessment of outcome | Follow-up long enough for outcomes to occur | Adequacy of follow-up of cohorts | Total Stars (out of 9) |
| --- | --- | --- | --- | --- | --- | --- | --- | --- | --- | --- |
| Palandri et al., 2024 | 0 | 0 | * | * | * | 0 | * | * | 0 | **5** |
| Huang et al., 2021 | * | 0 | * | * | * | 0 | * | * | * | **7** |
| Lee, Jr 2025 | * | * | * | * | * | * | * | * | * | **9** |
| Popova- Labachevska, M 2023 | 0 | 0 | * | * | 0 | 0 | * | * | * | **5** |
| Christen, M 2025 | * | 0 | * | * | 0 | 0 | * | * | * | **6** |
| Chen, CC 2024 | 0 | 0 | * | * | 0 | 0 | * | * | * | **5** |
| Chang, Yu-Sung 2025 | * | 0 | * | * | 0 | 0 | * | * | * | **6** |

**Supplementary Table S5 Quality Assessment using NIH Quality Assessment Tool for Observational Cohort and Cross-Sectional Studies**

<https://www.nhlbi.nih.gov/health-topics/study-quality-assessment-tools>

| Author & year | | Was the research question or objective in this paper clearly stated? | Was the study population clearly specified and defined? | Was the participation rate of eligible persons at least 50%? | Were all the subjects selected or recruited from the same or similar populations (including the same time period)? Were inclusion and exclusion criteria for being in the study prespecified and applied uniformly to all participants? | Was a sample size justification, power description, or variance and effect estimates provided? | For the analyses in this paper, were the exposure(s) of interest measured prior to the outcome(s) being measured? | Was the timeframe sufficient so that one could reasonably expect to see an association between exposure and outcome if it existed? | For exposures that can vary in amount or level, did the study examine different levels of the exposure as related to the outcome (e.g., categories of exposure, or exposure measured as continuous variable)? | Were the exposure measures (independent variables) clearly defined, valid, reliable, and implemented consistently across all study participants? | Was the exposure(s) assessed more than once over time? | Were the outcome measures (dependent variables) clearly defined, valid, reliable, and implemented consistently across all study participants? | Were the outcome assessors blinded to the exposure status of participants? | Was loss to follow-up after baseline 20% or less? | Were key potential confounding variables measured and adjusted statistically for their impact on the relationship between exposure(s) and outcome(s)? | Quality Rating (Good, Fair, Poor) |
| --- | --- | --- | --- | --- | --- | --- | --- | --- | --- | --- | --- | --- | --- | --- | --- | --- |
|  | Reeves, B 2025 | yes | yes | yes | yes | no | yes | yes | no | yes | no | yes | Cant determine | Cant determine | no | Fair |

**Supplementary Table S6: Additional Table: Safety Outcomes**

| **Study ID** | **≥Grade 3 Cytopenias (n, %) (anemia, leukopenia, neutropenia, thrombocytopenia)** | **≥Grade 3 Hepatotoxicity/Elevated Liver Enzymes (n, %)** | **≥Grade 3 Flu-like Symptoms (n, %)** | **Other Grade Adverse Events** | **Treatment-Related Mortality (n, %)** | **Discontinuation Rate (n, %)** | **Reasons for Discontinuation** |
| --- | --- | --- | --- | --- | --- | --- | --- |
| **(Barbui et al., 2021)**[7] | Neutropenia: •Ropeg: 4 (8%) •Phlebotomy: 0 | Hypertransaminasemia: •Ropeg: 1 (2%) •Phlebotomy: 0 | 0 | Neutropenia; Pruritus; Hypertransaminasemia; Flu-like symptoms; Asthenia; Skin symptom; Pain; Knee impingement syndrome; Thrombosis | 0 | Ropeg: 4 (8%) Phlebotomy: 2 (4%) | Ropeg: Intractable pruritus, increased transaminases, fatigue, withdrawal of consent Phlebotomy: Consent withdrawal |
| **(Gisslinger et al., 2020)**[8] | Thrombocytopenia: •Ropeg group: 3 (2%) •HU group: 5 (4%) Leucopenia: •Ropeg group: 3 (2%) •HU group: 6 (5%) Anemia: •Ropeg group: 1 (1%) •HU group: 2 (2%) | ALT increased: •Ropeg group: 5 (4%) •HU group: 0 AST increased: •Ropeg group: 3 (2%) •HU group: 0 Hepatic enzymes increased: •Ropeg group: 1 (1%) •HU group: 0 | Influenza-like: •Ropeg group: 2 (2%) •HU group: 2 (2%) | Thrombocytopenia; Leukopenia; Anemia; Fatigue; GGT increased; Headache; Diarrhea; Dizziness; ALT increased; Arthralgia; Hypertension; Nasopharyngitis; Nausea; AST increased; Asthenia; Platelet count decreased; Pain in extremity; Abdominal pain; Influenza; Urinary tract infection; Neutropenia; Cataract; WBC count decreased; Hepatic enzyme increased; Anxiety; Atrial fibrillation; Cholelithiasis; Hypocalcemia; Osteoarthritis; Depression; Blood lactate dehydrogenase increased; Platelet count increased; Stomatitis; Hypokalemia; Neck pain; Granulocytopenia; Psoriasis; Cardiac failure | Ropeg group: 0 HU group: 1 (0.7%) | Ropeg group: 11 (8%) HU group: 5 (4%) | Ropeg group: drug-related toxicity (thrombocytopenia in one patient, hypothyroidism in two patients, dyspnoea and pneumonitis in one patient, increased AST and ALT in one patient, microcytic anemia and anemia in one patient, and anxiety, psoriasis, depression, Sjögren's syndrome, and rheumatoid arthritis in one patient each) HU group: drug-related toxicity: thrombocytopenia and anaemia in one patient, and basal cell carcinoma, pyrexia, skin ulcer, and intolerance to HU in one patient each. |
| **(Mesa et al., 2025)**[10] | Neutrophil count decreased: 1 (1%) in Ropeg group WBC count decreased: 1 (1%) in Ropeg group Anemia: 0 in Ropeg group | ALT increased: 1 (1%) for Ropeg AST increased: 1 (1%) for Ropeg Hepatic function abnormal: 1 (1%) for Ropeg | Influenza-like illness: 1 (1%) for Ropeg | Beta-2 microglobulin urine increased; Weight loss; GGT increase; Pruritus; Alopecia; Anemia; Malaise; Fatigue; Urinary tract infection; Arthralgia; Myalgia; Diarrhea; Decreased appetite; Cough | Ropeg: 0 | Ropeg group: 9 (9%) | Due to treatment-emergent AEs: 5 (5%) Due to drug-related AEs: 4 (4%) |
| **(Huang et al., 2021)**[20] | Anemia: 2 Leukopenia: 1 Thrombocytopenia: 1 | 1 (11.1%) transaminitis | 0 | Depression; Alopecia; Bone pain; Neuropathy; Headache; Insomnia; Mucositis; Dizziness; Transaminitis | 0 | 2 (22.2%) | Disease progression: 1 to AML, 1 to secondary MF |
| **(Barbui et al., 2023)**[21] | Neutropenia: •EXP: 8 (9.2%) •Phl-O: 0 | Hypertransaminasemia: •EXP: 2 (2.3%) | 0 | Leukopenia; Neutropenia; Flu-like symptoms | 0 | EXP: 7 (8%) | Due to AE:  EXP: 7 (8%); Phl-O: 0 (0%) |
| **(Popova-Labachevska et al., 2023)**[22] | NR | NR | NR | Flu-like symptoms | 0 | NR | NR |
| **(Barbui et al., 2024)**[23] | Neutropenia: 1 (1.6%) | Increased transaminases: 1 (1.6%) | NR | Asthenia; Pruritus; Thyroid disorder; Metrorrhagia | NR | Ropeg arm: 21 (33%) Phl-O arm: 44 (70%) | Ropeg: Lack of response (n=12), Adverse Events (n=9), Withdrawal of consent (n=3) Phl-O: Lack of response (n=34), Withdrawal of consent (n=10) |
| **(Suo et al., 2024)**[24] | WBC decrease: 2 (4.1%) Neutrophil count decrease: 2 (4.1%) Lymphocyte count decrease: 1 (2.0%) | ALT increase: 1 (2.0%) GGT increase: 2 (4.1%) | NR | Hyperuricemia; Hypertriglyceridemia; ALT increase; AST increase; Leukopenia; GGT increase; Neutropenia; Asthenia; Proteinuria; Alopecia; Hepatic steatosis; Weight loss; Pneumonia; Myasthenia gravis | 0 | 3 (6.1%) | Personal reasons: 2 patients Adverse Event: 1 patient (Grade 2 myasthenia gravis) |
| **(Qin et al., 2024)**[25] | NR | NR | NR | Transaminase elevation; Leukopenia; Neutropenia; Asthenia | NR | NR | NR |
| **(Palandri et al., 2024)** [26] | 0 | 0 | 0 | Flu-like syndrome; Alopecia; Xerophthalmia; Creatinine increase; Skin rash; Migraine; Thyroiditis | 0 | 0 | None |
| **(Kirito et al., 2024)**[27] | NR | AST increased: 1 (3.7%) ALT increased: 1 (3.7%) | Pyrexia: 2 (7.4%) | Hypothyroidism; Beta-2 microglobulin urine increased | 0 | 1 (3.7%) | Prostate cancer (unrelated to drug): 1 |
| **(Reeves et al., 2025)**[28] | NR | NR | NR | Psychiatric disorders; Eye disorders; Gastrointestinal disorders; General disorders; Hepatobiliary disorders; Neoplasms; Skin disorders; Cardiac disorders | NR | NR | NR |
| **(Yoon et al., 2025)**[29] | Anemia: 1 (1%) | Hepatotoxicity: 1 (1%) | Fatigue: 1 patient (1%) Pyrexia: 1 patient (1%) | Alopecia; Hepatobiliary disorders; General disorders; Anemia; Hypertriglyceridemia; Bipolar disorder; Stable angina; Hepatotoxicity | 0 | 20 (20%) | TRAEs: 2; Disease Progression: 1; Other reasons (Physician decision, Withdrawal, Lost to follow-up, Protocol violation): 17 |
| **(Lee et al., 2025)**[30] | NR | NR | NR | Hepatotoxicity; Blood and lymphatic disorders; General disorders; Fatigue; Pruritus; Gastrointestinal disorders; Musculoskeletal disorders; Psychiatric disorders | NR | NR | NR |
| **(Chen et al., 2024)**[31] | Pancytopenia: 1 (4.8%) Neutropenia: 1 (4.8%) Anemia: 1 (4.8%) Thrombocytopenia: 1 (4.8%) | Transaminitis: 1 (4.8%) | 0 | Fatigue; Alopecia; Transaminitis; Musculoskeletal symptoms; Hypothyroidism; Depression; Insomnia; Diarrhea | 0 | 3 (14.3%) | Achievement of MMR and desire for pregnancy (1 patient), drug supply issue (1 patient), achievement of MMR (1 patient) |
| **(Christen et al., 2025)**[32] | 0 | 0 | 0 | Flu-like symptoms; Mood disturbances; Cytopenias; Dermatologic symptoms; Neurologic symptoms; Raynaud's phenomenon; Hypertension; Loss of appetite | 0 | 5 (25%) | Insufficient response (2), side effects (3) [restlessness, nausea, shortness of breath, gum pain, joint pain together with a persistent depressive mood, mild diarrhea, fatigue, headaches, and progressively worsening mood changes] |
| **(Chang et al., 2025)**[33] | Thrombocytopenia: 1 (1.6%) Leukopenia: 1 (1.6%) | Transaminitis: 1 (1.6%) Elevated GGT: 1 (1.6%) | 0 | Transaminitis; Fatigue; GGT increase; Alopecia; Headache; Thyroid disorders | 0 | 15 (23.4%) | Absence of response (38%), loss of response (13%), proceeding to HSCT (n=2), loss to follow-up (n=2), death (n=1), financial constraints (n=1), intolerable discomfort (n=1). Discontinuations were most frequent in MF (36%). |
| **(Inano et al., 2026)**[34] | 0 | 0 | 0 | Beta-2 microglobulin urine increased; Alopecia; AST increase; ALT increase; Anemia; Leukopenia; Pyelonephritis; Hypertriglyceridemia; Chronic obstructive pulmonary disease; Disseminated intravascular coagulation | 0 | 0 | NR |

**Abbreviations**: NR, not reported; AE, adverse event; ALT, alanine aminotransferase; AML, acute myeloid leukemia; AST, aspartate aminotransferase; EXP, experimental (ropeginterferon + phlebotomy arm); GGT, gamma-glutamyl transferase; HSCT, hematopoietic stem cell transplantation; HU, hydroxyurea; MF, myelofibrosis; MMR, major molecular response; NR, not reported; Phl-O, phlebotomy-only; TRAE, treatment-related adverse event; WBC, white blood cell.
